# Aggregate Filamentous Growth Responses in Yeast

**DOI:** 10.1128/mSphere.00702-18

**Published:** 2019-03-06

**Authors:** Jacky Chow, Heather M. Dionne, Aditi Prabhakar, Amit Mehrotra, Jenn Somboonthum, Beatriz Gonzalez, Mira Edgerton, Paul J. Cullen

**Affiliations:** a Department of Biological Sciences, State University of New York at Buffalo, Buffalo, New York, USA; b Department of Oral Biology, State University of New York at Buffalo, Buffalo, New York, USA; Yonsei University

**Keywords:** MAP kinase, Rho GTPase, cell differentiation, cell polarity, cell shape, collective cellular responses, fungal morphogenesis, fungal pathogenesis, quasisociality

## Abstract

Many fungal species, including pathogens, undergo a morphogenetic response called filamentous growth, where cells differentiate into a specialized cell type to promote nutrient foraging and surface colonization. Despite the fact that filamentous growth is required for virulence in some plant and animal pathogens, certain aspects of this behavior remain poorly understood. By examining filamentous growth in the budding yeast Saccharomyces cerevisiae and the opportunistic pathogen Candida albicans, we identify responses where cells undergo filamentous growth in groups of cells or aggregates. In S. cerevisiae, aggregate invasive growth was regulated by signaling pathways that control normal filamentous growth. These pathways promoted aggregation in part by fostering aspects of microbial cooperation. For example, aggregate invasive growth required cellular contacts mediated by the flocculin Flo11p, which was produced at higher levels in aggregates than cells undergoing regular invasive growth. Aggregate invasive growth was also stimulated by secreted enzymes, like invertase, which produce metabolites that are shared among cells. Aggregate invasive growth was also induced by alcohols that promote density-dependent filamentous growth in yeast. Aggregate invasive growth also required highly polarized cell morphologies, which may affect the packing or organization of cells. A directed selection experiment for aggregating phenotypes uncovered roles for the fMAPK and RAS pathways, which indicates that these pathways play a general role in regulating aggregate-based responses in yeast. Our study extends the range of responses controlled by filamentation regulatory pathways and has implications in understanding aspects of fungal biology that may be relevant to fungal pathogenesis.

**IMPORTANCE** Filamentous growth is a fungal morphogenetic response that is critical for virulence in some fungal species. Many aspects of filamentous growth remain poorly understood. We have identified an aspect of filamentous growth in the budding yeast Saccharomyces cerevisiae and the human pathogen Candida albicans where cells behave collectively to invade surfaces in aggregates. These responses may reflect an extension of normal filamentous growth, as they share the same signaling pathways and effector processes. Aggregate responses may involve cooperation among individual cells, because aggregation was stimulated by cell adhesion molecules, secreted enzymes, and diffusible molecules that promote quorum sensing. Our study may provide insights into the genetic basis of collective cellular responses in fungi. The study may have ramifications in fungal pathogenesis, in situations where collective responses occur to promote virulence.

## Abstract (precis)

Filamentous growth is a fungal morphogenetic response that is critical for virulence in some fungal species. Many aspects of filamentous growth remain poorly understood. We have identified an aspect of filamentous growth in the budding yeast Saccharomyces cerevisiae and the human pathogen Candida albicans where cells behave collectively to invade surfaces in aggregates. These responses may reflect an extension of normal filamentous growth, as they share the same signaling pathways and effector processes. Aggregate responses may involve cooperation among individual cells, because aggregation was stimulated by cell adhesion molecules, secreted enzymes, and diffusible molecules that promote quorum sensing. Our study may provide insights into the genetic basis of collective cellular responses in fungi. The study may have ramifications in fungal pathogenesis, in situations where collective responses occur to promote virulence.

## INTRODUCTION

Many fungal species are capable of undergoing filamentous growth, where cells change their shape and growth pattern in response to nutrient limitation and other stresses. Many plant and animal pathogens undergo filamentous growth during part or all of their life cycles (1–3). In the human opportunistic pathogen Candida albicans, filamentous growth is required for virulence (4, 5). In that organism, cells form filaments to attach to and penetrate host tissues (6–8). A great diversity in filamentation responses has been observed in fungal species. For example, the plant pathogen Rhizoctonia solani makes an infection cushion across the host surface followed by the reorientation of hyphae to penetrate the plant epidermis (9). How groups of cells coordinate filamentous growth responses is not entirely clear. Many fungal species also engage in biofilm/mat formation, where cells grow in mats or groups (1, 10–13). Filamentous growth and biofilm/mat formation are related responses that occur in complex relationships during infection (14, 15). Other key facets of fungal pathogenicity also involve changes in genome stability (16) and cell surface variegation (17, 18), which create variation on the fungal cell surface to evade the host’s immune system. The interrelated aspects of fungal community development are common among free-living and pathogenic fungal species (19).

The budding yeast Saccharomcyes cerevisiae also undergoes filamentous growth and has been used as a model to understand the genetic and molecular basis of this behavior (20, 21). In response to carbon or nitrogen limitation, yeast of certain strain backgrounds (Σ1278b was used in this study) differentiate into the filamentous cell type (22). Among the readily observable changes that occur during filamentous growth are an elongated cell shape and a distal-unipolar budding pattern. In addition, filamentous cells remain physically connected after cytokinesis, which results in the formation of chains of cells or filaments. As a result of these and other changes, cells expand outward from colony centers across surfaces (pseudohyphal growth), or downward into surfaces (invasive growth). Invasive growth has been mainly studied in haploids by the plate-washing assay (PWA), where cells on the surface of a colony are removed by washing with a gentle stream of water to reveal invaded cells (23). Invasive growth and pseudohyphal growth are related aspects of filamentous growth that share common elements yet also have unique features.

Filamentous growth in yeast is induced by stimuli that are sensed and relayed by signal transduction pathways. The limitation of fermentable carbon sources, like glucose, induces a mitogen-activated protein kinase pathway (fMAPK) (23–25). Specifically, growth in nonpreferred carbon sources causes underglycosylation and subsequent cleavage of the signaling mucin Msb2p (26–29). Processing and release of the inhibitory extracellular glycodomain of Msb2p lead to activation of a MAPK pathway that is controlled by the Rho-type GTPase Cdc42p, a master regulator of polarity and signaling (30). Cdc42p-dependent MAPK activation culminates in phosphorylation of the MAP kinase Kss1p (20). Kss1p regulates a suite of transcription factors (Ste12p and Tec1p [31], Msa1p and Msa2p [32], and the repressor Dig1p [33]) that control target gene expression to bring about the filamentous cell type.

The fMAPK pathway is one of many pathways and protein complexes that regulate filamentous growth (34–37). Another major regulatory pathway that has been well characterized is the Ras-cAMP-PKA (RAS) pathway. In that pathway, a seven-transmembrane receptor binds to glucose and other sugars, called Gpr1p. Gpr1p and an associated heterotrimeric G-protein (38–40) regulate the major nutrient-regulatory GTPase Ras2p (41), which activates adenylate cyclase to produce cAMP (41–43). cAMP binds to the regulatory subunit of protein kinase A (PKA; there are three Tpks in yeast) (44–46). A specific Tpk, Tpk2p (47, 48), controls the activity of the transcription factor Flo8p. Other regulators of filamentous growth include the major AMP-dependent kinase (AMPK) Snf1p (49–52), the retrograde mitochondria-to-nucleus (RTG) pathway (53–56), the pH-sensitive Rim101 pathway (RIM101) (57–62), and the pathway that controls phosphate utilization and many other functions, which is regulated by the cyclin-dependent kinase Pho85p (63, 64) (J. Chow, I. Starr, S. Jamalzadeh, O. Muniz, A. Kumar, O. Gokcumen, D. M. Ferkey, and P. J. Cullen, submitted for publication). These and other pathways operate in an integrated network, where multiple pathways control each other’s activities and targets (54, 65–69) (Chow et al., submitted). One specific point of integration among the pathways is the *FLO11* promoter (70–72). Flo11p is a glycosylphosphatidylinositol (GPI)-anchored mucin-type cell adhesion molecule (73–77) that regulates filamentous growth (17, 72, 78) and biofilm/mat formation (11).

Despite the fact that many proteins and pathways that regulate filamentous growth have been identified and characterized, certain aspects of the response remain mysterious. For example, invasive growth by the PWA can reveal a diversity of patterns. The molecular basis of the different patterns is not clear. Likewise, patterns of pseudohyphal growth can be complex and are not well understood (22). By examining different patterns of filamentous growth in several fungal species, we characterize here aspects of the response where cells interact collectively in groups or aggregates. We focused on aggregate invasive growth, which required the same regulatory pathways as regular invasive growth and may be an extension of the normal filamentation response. Aggregate invasive growth might result from cooperation among groups of cells. Cells in aggregates showed increased Flo11p levels, and Flo11p was required for the formation of invasive aggregates. Aggregate invasive growth was also stimulated by alcohols that are known to induce density-dependent responses (79, 80) and by secreted enzymes, like invertase, which promote cooperation by producing metabolites (i.e., shared goods) that can be shared among individuals in a community (81–83). Therefore, aggregate invasive growth may result from the physical interactions and cooperation among groups of filamentous cells.

Directed selection approaches have identified new behaviors in S. cerevisiae, including regulatory proteins that contribute to multicellular-type responses (84–87). Directed selection experiments to enrich for aggregate phenotypes showed roles for the fMAPK and Ras pathways, which may indicate a general function for these pathways in regulating group responses in yeast. Some phenotypes were independent of these pathways, indicating that other pathways may also be involved. The genetic changes underlying several of the aggregating isolates included genes that impact fMAPK pathway activity, that control aspects of Cdc42p-dependent bud-site selection, and that regulate cell separation. Collectively, our findings characterize regulatory mechanisms that govern the collective interactions among cells during a fungal differentiation response. Our findings may be relevant to studies of pathogenic fungi that may employ collective responses during pathogenesis.

## RESULTS

### Aggregate filamentous growth responses in baker’s yeast and Candida albicans.

**FIG 1:**
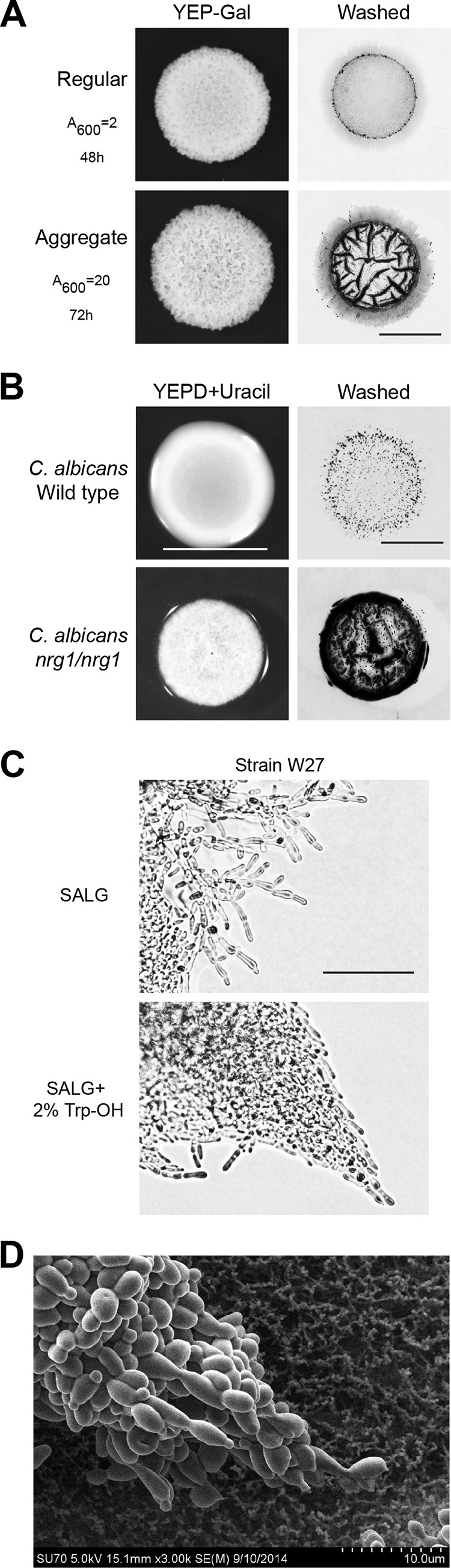
Aggregate-type filamentous growth responses in yeast. (A) PWA of wild-type S. cerevisiae (PC6021) spotted on YEP-Gal to induce regular invasive growth (*A*_600_ OD = 0.2, 48 h) or strongly promote aggregate invasive growth (*A*_600_ OD = 20, 72 h; plate dried for 10 days). Colony (YEP-Gal) and washed plates (Washed) are shown. Bar, 0.5 cm. (B) PWA of wild-type and *nrg1/nrg1* C. albicans spotted on YEPD + URA. Colony (YEPD + URA) and washed plates (Washed) are shown. Bar, 0.5 cm. (C) Pseudohyphal aggregates formed at the colony perimeter by the W27 (diploid) S. cerevisiae strain on SLAD medium. Addition of Trp-OH (500 µM) induced assembly of filaments into large pointed aggregates. (D) SEM of pseudohyphal filament formed by p*GAL-FLO11* S. cerevisiae strain (PC2712) grown on nitrocellulose.

We noticed that invasive growth and psedohyphal growth in yeast occurred in different patterns. Standard conditions for invasive growth (YEP-Gal, starting cell concentration *A*_600_ = 2, 48 h of incubation at 30°C) showed a heterogeneous pattern of invading cells. Dark “divots” of invaded cells at the perimeter surrounded a light central region of regular invasive growth (Fig. 1A, Regular). Increasing the concentration of cells (*A*_600_ = 20) and incubation time (72 h) made the divots more pronounced (Fig. 1A, Aggregate). As seen below, the size and shape of divots were also influenced by pH, nutrient levels, and medium type, which we refer to here as invasive aggregates. Invasive aggregates were also produced by natural isolates of S. cerevisiae (see below) and the opportunistic pathogen Candida albicans. For C. albicans, a speckled pattern was observed in wild-type cells that was exaggerated in hyperfilamentous mutants (Fig. 1B). The different patterns might reflect different aspects of the filamentous growth response.

We also found that pseudohyphal growth in S. cerevisiae occurred not only in single chains of cells but also in complex patterns. Pseudohyphal aggregates were abundant in natural isolates of yeast and could be induced by established triggers of filamentous growth (Fig. 1C, tryptophol, Trp-OH; see below). Although pseudohyphal aggregates have not been explicitly defined, this type of response is evident in data published for *Cryptococcus* (88), Candida albicans (89), Candida glabrata (90), and Schizosaccharomyces pombe (91). Aggregate pseudohyphal growth was also apparent in hyperfilamentous mutants, like those overproducing the adhesion molecule Flo11p. Scanning electron microscopy (SEM) highlighted the interactions of cells that can occur during this type of growth (Fig. 1D). Below we also show that groups of filamentous cells also aggregated in liquid culture. Thus, we became interested in understanding how yeast cells collectively organize during filamentous growth-type responses.

### Yeast can undergo aggregate invasive growth.

Aggregate invasive growth and aggregate pseudohyphal growth are probably different responses, although they may share some common elements. We focused on aggregate invasive growth for the following reasons. (i) Aggregate invasive growth occurred under specific environmental conditions, which indicated that the response was highly regulated. A potential concern was that aggregates occurred as a physical effect of growing large amounts of cells in colonies for long periods of time. However, aggregates did not form due to gravity, as their formation occurred on inverted plates with colonies growing upside down. (ii) Aggregate invasive growth occurred in multiple species, including S. cerevisiae and C. albicans. (iii) Preliminary experiments showed that aggregate invasive growth was regulated by signaling pathways and target genes that control aspects of the filamentous growth response. These observations supported the hypothesis that the response was highly regulated. (iv) Aggregate invasive growth occurred in haploid S. cerevisiae cells of the Σ1278b background, which facilitated genetic analysis of the response.

**FIG 2:**
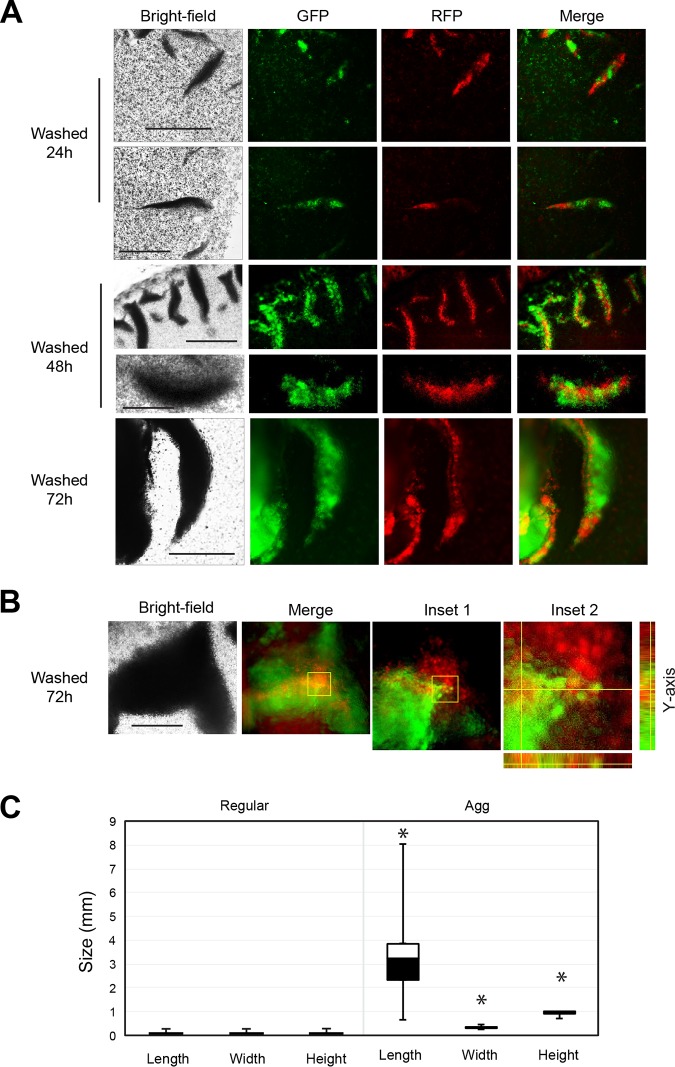
S. cerevisiae forms invasive aggregates. (A) Microscopic examination of aggregates. Red (PC6581) and green (PC6733) fluorescent cells were grown separately in liquid YEPD for 16 h, mixed in a 1:1 ratio, and spotted on YEP-Gal agar medium at the aggregate-inducing concentration (*A*_600_ OD = 20). Colonies were grown for 24 h (bars, 300 μm), 48 h (bar, 100 μm [top]; bar, 300 μm [bottom]), or 72 h before PWA (bar, 100 μm). The 72-h image shows overall pointed shape and composition of a section of an invasive aggregate. Shown are bright-field images of aggregates and merged GFP and rhodamine fluorescent channel images. (B) Microscopic examination of aggregate with *z* axis analysis (bar, 100 μm). Shown are bright-field, merged, and inset images. Inset 1 is of selection from merged image. Inset 2 is of selection from inset 1. Inset 2, right, shows *z* slices in the *y* axis, and the bottom shows *z* slices in the *x* axis. (C) Box-and-whisker plots, showing the dimensions of invasive filaments undergoing regular invasive growth and dimensions of invasive aggregates at 72 h. Asterisk, *P* value of <0.05.

The different patterns of invasive growth were examined by microscopy. Microscopic examination showed that light regions were composed of small groups of cells (<10 cells) undergoing regular invasive growth as filaments (Fig. 2A, Bright-field). By comparison, the divots were composed of dense groups of many cells (Fig. 2A, Bright-field). Aggregates might form by the growth and division of cells from a single progenitor or from groups of cells that come from multiple progenitors. To distinguish between these possibilities, the composition of invasive aggregates was examined. Fluorescent (RFP and GFP) strains were constructed and mixed. Invasive scars were examined by fluorescence microscopy. This experiment showed that aggregates were composed of differently labeled groups of cells (typically in a 1:1 ratio [0.94 ± 0.05, *n* = 10]). Since no single-color aggregates were observed (Fig. 2A, Merge), we conclude that invasive aggregates were formed from different groups of cells. The chimeric nature of aggregates was visible in small (24-h), medium (48-h), and large (72-h) aggregates (Fig. 2A, Merge). At 72 h, aggregates were composed of many groups of cells, which was evident by analysis of the fluorescent patterns at different magnifications and through the *z* axis of an aggregate (Fig. 2B).

Surface penetration during invasive growth allows cells to enter new environments, which is thought to promote foraging for nutrients (26). During regular invasive growth, cells formed chains of filaments composed of 5 to 10 cells that penetrated 25 to 50 μm below the surface (Fig. 2C, Regular, Height) (22, 26). By comparison, invasive aggregates produced macroscopic scars that were visible to the naked eye. The median dimensions of invasive aggregates were 3.2 mm long by 0.3 mm wide by 0.9 mm deep (Fig. 2C). At 72 h, a typical aggregate was composed of approximately 4.8 × 10^7^ cells, based on aggregate dimensions of a half-ellipsoid shape and the volume of a yeast cell as 37 μm^3^ (92). The 20-fold increase in surface penetration by aggregates may benefit nutrient foraging and range expansion into new environments. Furthermore, the pointed shape of aggregates would be expected to promote penetration into surfaces (93).

To learn more about how aggregates form, aggregate biogenesis was examined in growing colonies. Time-lapse photography showed that aggregates first appeared at a specific point early in colony development (see Fig. S1A, 12 h, in the supplemental material). Thus, aggregate invasive growth is not a result of colony growth or aging. Aggregates were initially detected above the agar surface (Fig. S1B). This result shows that aggregates do not form in response to imperfections in the agar surface. Aggregate formation may be related to other adhesion-dependent responses in yeast, including complex colony morphology (94) and biofilm/mat growth (11). Indeed, colony ruffles above the agar surface formed part of the structure of invasive aggregates. This was determined by dripping water onto the colony surface, which caused the colony/mat of connected cells to flip over and reveal the underside of the colony (Fig. S1C). The fact that colony ruffling contributes to aggregate formation may have been missed in other studies, because over time the ruffled pattern on the surface changes and does not match the invasive pattern. Therefore, aggregates are macroscopic structures that are composed of many groups of cells coming together, in part by colony pattern formation.

10.1128/mSphere.00702-18.1 FIG S1 Aggregate development. (A) An *hsl7*Δ strain grown on YEP-Gal agar media at 22°C photographed at the indicated times. PWA performed at 48 h. Inset (right) is of selection (left). (B) Confocal microscopy of early aggregates (10 h). Image series shows cells within the focal plane at the indicated depths beneath the surface. (C) An *hsl7*Δ strain grown on YEP-Gal agar medium at 30°C. Shown are the colony at 48 h, the underside of the colony photographed through a clear petri plate, and drip wash after dripping water near the colony to lift the colony off the agar surface. Download FIG S1, PDF file, 1.5 MB. Copyright © 2019 Chow et al. 2019 Chow et al. This content is distributed under the terms of the Creative Commons Attribution 4.0 International license.

### Signaling pathways that control filamentous growth are required for aggregate invasion.

**FIG 3:**
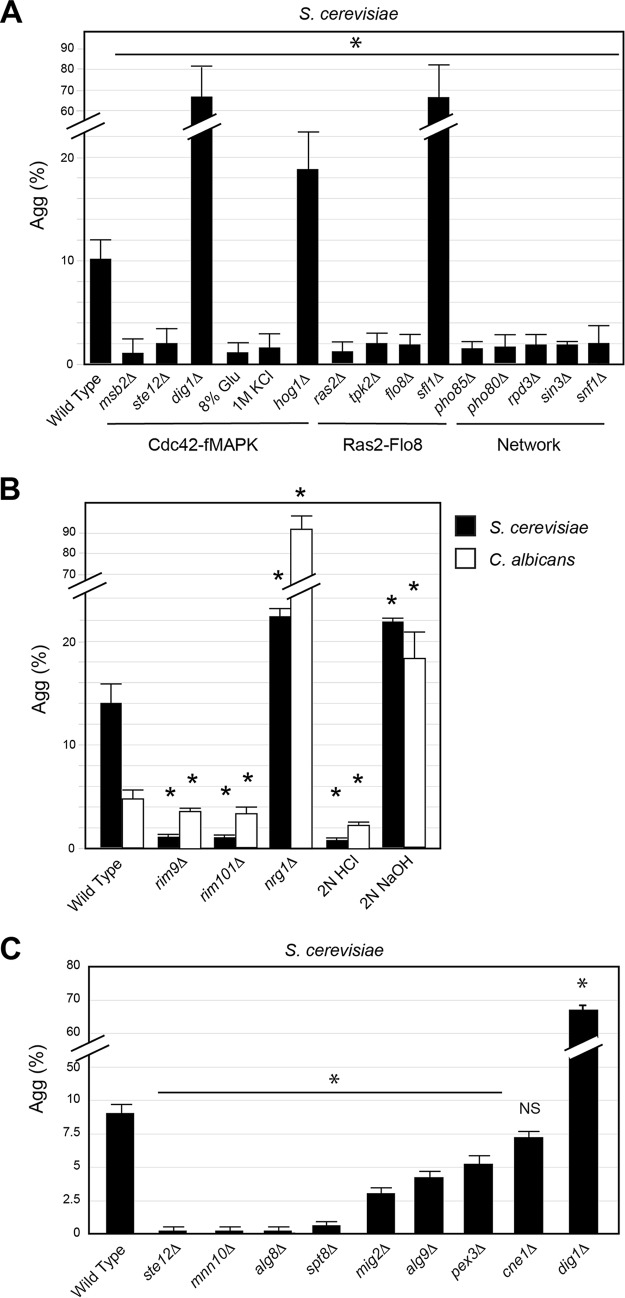
Role of filamentous growth regulatory pathways in regulating aggregate invasive growth. (A) Bar graph showing the average amount of aggregates in a scar expressed as a percentage of the total colony surface area [Agg (%)] for deletion mutants of established filamentous growth regulators. Error bars represent standard error. Asterisk, *P* value of <0.05. (B) Bar graph showing the effect of pH on and the role of the pH-sensitive Rim101 pathway in invasive aggregate formation in *S. cerevisiae* (black) and C. albicans (white). Error bars represent standard error. Asterisk in comparison to wild type, *P* value of <0.05. (C) Bar graph showing the average amount of aggregates in a scar expressed as a percentage of the total colony surface area [Agg (%)] for deletion mutants identified by a C. albicans deletion screen (Table S2).

To evaluate the genetic basis of aggregate invasive growth, a method was developed to quantitate the degree of aggregate formation. ImageJ analysis was used to measure aggregates in an invasive scar that was expressed as a percentage of the total colony surface area, which took into account aggregate size (% Agg). For wild-type cells at 48 h on YEP-Gal (*A*_600_ = 20), approximately 10% of invasive growth occurred in the aggregate pattern (Fig. 3A). This method was used to compare aggregate invasive growth under different conditions and in mutants lacking filamentation regulatory pathways.

One pathway that regulates filamentous growth is the fMAPK pathway (Fig. S2A) (23, 25, 69, 95, 96). A mutant that lacked the mucin-type sensor that functions at the head of the fMAPK pathway failed to form aggregates (Fig. 3A, *msb2*Δ; see Fig. S2B for the raw data) (25), as did a mutant that lacked one of the transcription factors for the pathway (Fig. 3A, *ste12*Δ) (23). Similarly, a mutant that lacked a transcriptional repressor of the fMAPK pathway made better aggregates (Fig. 3A, *dig1*Δ) (97). The fMAPK pathway is activated by growth in nonfermentable carbon sources (26, 29). High levels of glucose, which repress the activity of the fMAPK pathway, inhibited aggregate formation (Fig. 3A, 8% Glu). The high-osmolarity glycerol response (HOG) pathway responds to osmotic stress and also functions to inhibit the fMAPK pathway (98). The addition of salt to the medium inhibited aggregate invasive growth (Fig. 3A, 1 M KCl), and a mutant that lacked a functional HOG pathway made better aggregates (Fig. 3A, *hog1*Δ). Thus, the fMAPK pathway is required for aggregate invasive growth.

10.1128/mSphere.00702-18.2 FIG S2 Role of signaling pathways in regulating aggregate invasive growth. (A) The fMAPK pathway (20). Glucose limitation causes Msb2p-dependent activation of the fMAPK pathway that regulates invasive growth. Green denotes key positive regulators; red denotes key negative regulators, including the HOG pathway MAP kinase Hog1p. At left, generic regulators are shown in gray. *CDC42* is an essential gene and was therefore not tested. (B) The PWA. The indicated strains were grown under aggregate-inducing conditions (10-μl aliquot of cells with an OD *A*_600_ of 20, for 48 h, at 30°C, on YEP-Gal medium) except where otherwise described. 8% Glu refers to YEP + 8% glucose; 1 M KCl refers to YEP-Gal + 1 M KCl (cells were grown for 7 days). Bar, ∼1 cm. (C) The Ras-cAMP-PKA pathway (188). Green, positive regulators; red, negative regulators. (D) The PWA. Strains grown under aggregate-inducing conditions. Bar, ∼1 cm. (E) Multiple pathways regulate filamentous growth in an integrated network (54). Representative positive regulators of filamentous growth were tested. This panel is adapted from reference 54 with permission of the publisher. (F) The PWA. Strains grown under aggregate-inducing conditions. Bar, ∼1 cm. Download FIG S2, PDF file, 0.2 MB. Copyright © 2019 Chow et al. 2019 Chow et al. This content is distributed under the terms of the Creative Commons Attribution 4.0 International license.

Another major regulator of filamentous growth in yeast and other fungi is the RAS pathway (Fig. S2C) (22, 37, 70, 94, 99, 100). Cells lacking the GTPase Ras2p (Fig. 3A, *ras2*Δ; Fig. S2D), the filamentation-specific protein kinase A subunit Tpk2p (*tpk2*Δ), or the transcription factor Flo8p (70, 101) were defective for aggregate invasive growth (Fig. 3A, *flo8*Δ). The *tpk2*Δ mutant showed a minor defect, which may indicate that other Tpks or other proteins might also be involved. Likewise, cells lacking the negative regulator Sfl1p (102) made better aggregates (Fig. 3A, *flo8*Δ). Sfl1p functions to regulate Flo11p levels, which can result in variegation of Flo11p among cells in the population (17, 18). Aggregates may form due to reduced variegation in Flo11p levels among cells in the population. Although this is a possibility, we did not observe any obvious variegation of Flo11p-HA levels in normal cells or cells in aggregates. What we saw instead was a uniform increase in the amount of Flo11p-HA in cells derived from aggregates (see below). This correlated with the fact that cells in normal filaments were uniformly less elongated than cells in aggregates (see below). Collectively, the results indicate that the RAS pathway is also required for aggregate formation.

In addition to the fMAPK and RAS pathways, filamentous growth is also regulated by multiple other pathways, many of which operate in a coordinated network (54, 65–68). An example of the network can be seen in Fig. S2E (adapted from reference 54). A subset of the pathways in the network was tested. The cyclin-dependent kinase Pho85p and alternative cyclin Pho80p (64) have previously been identified as regulators of filamentous growth (54). Both of these proteins were required for aggregate invasion (Fig. 3A, *pho85*Δ and *pho80*Δ; Fig. S2F). Chromatin remodeling proteins Rpd3p and Sin3p also regulate filamentous growth (65, 103) and were required for aggregate invasion (Fig. 3A, *rpd3*Δ and *sin3*Δ). Snf1p, the major AMP-dependent protein kinase that regulates the derepression of glucose-repressed genes (104) and is required for filamentous growth (26, 103, 105, 106), was also required for aggregate invasion (Fig. 3A, *snf1*Δ). These results demonstrate that aggregate invasive growth is a highly regulated process that is controlled by key regulators of filamentous growth.

### Common regulators of aggregate invasive growth in C. albicans and S. cerevisiae include the Rim101 pathway and SAGA.

The fact that C. albicans also formed invasive aggregates (Fig. 1B; also see Fig. S3A) led us to ask whether common regulatory pathways controlled aggregate invasive growth in both species. To identify regulators of aggregate invasive growth that were conserved in both species, 1,186 C. albicans mutants in partially redundant collections that contained 928 unique gene disruptions (107–113) were examined for phenotypes in aggregate invasive growth. This approach identified 135 genes (Table S2). Many of the genes were common between C. albicans and S. cerevisiae, including genes that regulate the C. albicans Cek1p pathway, which is homologous to the S. cerevisiae fMAPK pathway (114). The screen also identified genes that comprise the Rim101 pathway, which is a pH-sensitive pathway in both species (58–60, 115–117). The Rim101 pathway was required for aggregate invasive growth in S. cerevisiae and C. albicans (Fig. 3B, *rim9*Δ, *rim101*Δ; Fig. S3B), and loss of the negative regulator Nrg1p led to an increase in aggregate invasion in both species (Fig. 3B, *nrg1*Δ). High pH stimulated aggregate invasive growth (Fig. 3B), and low pH suppressed it in both species (Fig. 3B). The C. albicans screen was somewhat less effective at uncovering the expected mutations, as was anticipated (Table S2). This may be because of functional redundancy in adhesion molecules and/or regulatory pathways. Alternatively, it might be because C. albicans forms true hyphae, which might impact aggregate formation. Finally, the conditions tested may not have been optimal for identification of regulators of aggregate formation. Therefore, common signaling pathways regulate aggregate invasive growth in S. cerevisiae and C. albicans.

10.1128/mSphere.00702-18.3 FIG S3 The Rim101 pathway and new regulators of aggregate invasive growth that are shared in C. albicans and S. cerevisiae. (A) C. albicans grown at the indicated starting concentrations on YEPD + 50 μM uridine. (B) PWA. C. albicans from the Nobile 2009 library was on YEPD + 50 μM uridine for 48 h at 30°C. Wild-type C. albicans was also grown with the indicated pH spot. Bar, 1.25 cm. S. cerevisiae strains were grown under standard aggregate-inducing conditions. Wild-type S. cerevisiae was also grown with the indicated pH spot. Bar, 1.25 cm. (C) Pie chart of regulators of aggregate invasion identified by the genetic screen. See Table S2 for details. For a subset of targets identified by the C. albicans screen, homologous genes were knocked out in the S. cerevisiae wild-type strain (PC538). (D) PWA of a subset of mutants is shown. Download FIG S3, PDF file, 0.5 MB. Copyright © 2019 Chow et al. 2019 Chow et al. This content is distributed under the terms of the Creative Commons Attribution 4.0 International license.

The screen also identified genes that impacted aggregate invasive growth but have not been previously connected to known filamentation regulatory pathways, which fell into several functional categories (Table S2; Fig. S3C). To determine whether these genes were general regulators of aggregate invasive growth in both species, mutants containing the homologous gene deletions were examined in S. cerevisiae. Several genes regulated aggregate invasive growth in both species (Fig. 3C; Fig. S3D). One of these was Spt8p (Fig. 3C, *spt8*Δ), a component of the SAGA chromatin-remodeling complex (118). Spt8p also regulated normal invasive growth and in a parallel study was found to regulate colony ruffling and mat formation (Chow et al., submitted). Therefore, evolutionarily conserved signaling pathways (e.g., MAPK and RIM101) and transcriptional regulatory complexes (e.g., SAGA) regulated aggregate invasive growth in C. albicans and S. cerevisiae. Although it is possible that genes or pathways specifically control aggregate invasive growth, all of the genes that regulate aggregate invasive growth tested here were also required for normal invasive growth. Collectively, these results suggest that aggregate invasive growth is an extension of the normal invasive growth response.

### The cell adhesion molecule Flo11p is required for aggregate invasive growth.

**FIG 4:**
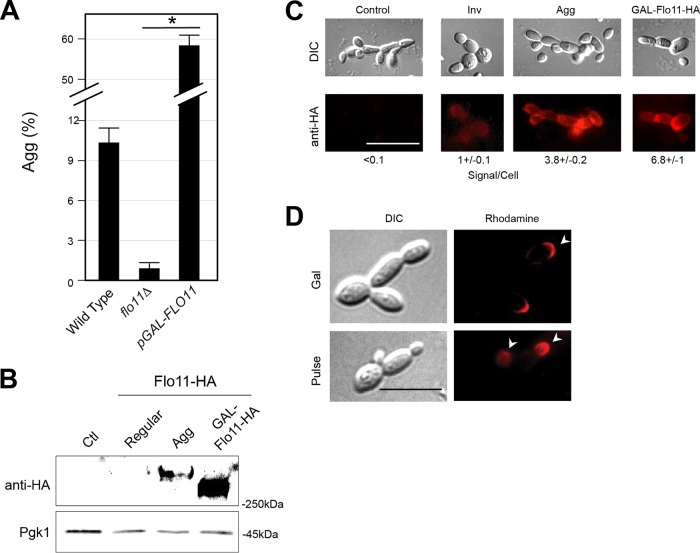
The cell adhesion molecule Flo11p is required for aggregate invasive growth. (A) Aggregation [Agg (%)] of the indicated strains. (B) Immunoblot showing the amount of Flo11p-HA present in invaded cells: negative-control (Ctl, untagged strain), regular invasive (Regular), and aggregate-enriched (Agg) cells expressing Flo11p-HA and cells overproducing Flo11-HA (GAL-Flo11p-HA). Pgk1p was a control for protein loading. (C) Immunofluorescence showing the amount of Flo11p-HA present in invaded cells: negative-control (Control, untagged strain), regular invasive (Inv), and aggregate-enriched (Agg) cells expressing Flo11p-HA and cells overproducing Flo11p-HA (GAL-Flo11p-HA). Rhodamine exposure times were identical for regular and aggregate cells, with a shorter exposure for GAL-Flo11p-HA. Fluorescent signal per cell was normalized to average signal per cell of normal invasive filament. Bar, 10 μm. (D) Immunofluorescence showing distribution of Flo11p-HA in an overexpression mutant, GAL-Flo11p-HA, or under the endogenous promoter, Flo11p-HA. Pulse, GAL-Flo11p-HA strain following 4-h pulse with galactose. Gal, Flo11p-HA strain following 4-h pulse with galactose. Arrowheads mark the distal tip of daughter cells. Bar, 5 μm.

The major signaling pathways that regulate filamentous growth, along with many other proteins and pathways (37), control differentiation to the filamentous cell type. One change is increased cell adhesion by transcriptional induction of the gene encoding the adhesion molecule Flo11p (Fig. S4A). Flo11p was required for aggregate invasive growth (Fig. 4A, *flo11*Δ; Fig. S4B), and cells overproducing Flo11p made better aggregates (Fig. 4A*, pGAL-FLO11*; Fig. S4B). Additionally, while wild-type cells achieved aggregate invasive growth at high cell concentrations (Fig. 1A, Regular versus Aggregate), aggregate invasive growth occurred at low cell concentrations when overproducing Flo11p (Fig. S4B).

10.1128/mSphere.00702-18.4 FIG S4 *FLO11* overexpression bypasses density dependency for aggregate invasive growth. (A) Model showing important behavioral changes during filamentous growth regulated by fMAPK: adhesion, secreted enzymes, distal polarity, and apical growth. Green text shows a subset of key target genes. (B) PWA of indicated strains (wild-type, p*GAL-FLO11*, *sfl1*Δ, and *flo11*Δ), at the indicated concentrations, for 24 or 48 h. Agg (%) is shown at right. Download FIG S4, TIF file, 2.8 MB. Copyright © 2019 Chow et al. 2019 Chow et al. This content is distributed under the terms of the Creative Commons Attribution 4.0 International license.

To define how aggregates might differ from normal invasive cells, we developed a method to enrich for cells in aggregates. Immunoblot analysis of cells expressing a functional epitope-tagged version of Flo11p (Flo11p-HA) showed that cells in aggregates produced more Flo11p-HA than cells undergoing regular invasive growth (Fig. 4B). Overexpression of Flo11p-HA, as a control, led to a lower-molecular-weight band, which may be due to underglycosylation of the overproduced protein, which has been seen for another large glycoprotein, Msb2p (29). Immunofluorescence analysis showed that cells in aggregates produced more Flo11p-HA on the cell surface than regular filamentous cells (Fig. 4C). To further explore the localization patterns of Flo11p, two experiments were performed. In one experiment, cells expressing Flo11p-HA from its endogenous promoter were shifted from glucose to galactose, which induces *FLO11* expression (75). The result was an enrichment of Flo11p-HA on the tips and sides of cells (Fig. 4D, Gal). In a second experiment, Flo11p-HA was induced by a galactose-driven promoter, which also showed Flo11p-HA at highly polarized sites (Fig. 4D, Pulse). The fact that Flo11p is found at the tips and sides of cells in aggregates might begin to account for the collective organization of cells during aggregate formation.

### Distal-unipolar budding and cell shape contribute to aggregate formation.

**FIG 5:**
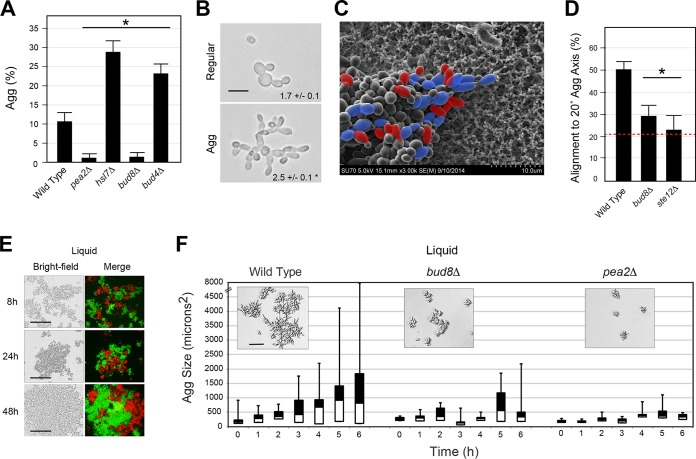
Distal-pole budding and cell elongation are critical for aggregate invasive growth. (A) Aggregation [Agg (%)] of budding and elongation mutants. Asterisk, *P* value of <0.05. (B) Bright-field image of cells scraped from regular invasive and aggregate-enriched cells. Values, length-to-width ratio. *n* = 83 aggregate cells, 69 normal filament cells. Asterisk, *P* value of <0.01. Bar, 5 μm. (C) Example of pseudohyphal aggregate of *pGAL-FLO11* strain on nitrocellulose by SEM at 3,000×. Blue, cell axis within 20° of aggregate axis; red, cell axis outside 20° of aggregate axis. (D) Alignment of *pGAL-FLO11*, *pGAL-FLO11 bud8*Δ, and *pGAL-FLO11 ste12*Δ cells to 20° of the major axis of the aggregate (%). Asterisk, *P* value of <0.02 compared to wild type. Red line, predicted alignment of randomly oriented cells. (E) Examples of mixed fluorescent red and green wild-type cells grown in liquid YEP-Gal medium for the indicated times (bar, 50 μm). (F) Box-and-whisker plot showing aggregate size (in µm^2^) at the indicated times for the strains tested. Cells were grown for 16 h in YEPD medium and transferred to YEP-Gal medium for the indicated times. Differences between wild-type and mutant are significant at *t* = 6 h, *P* < 0.05. Insets show representative examples of aggregates. Bar, 20 μm.

In addition to changes in cell adhesion, filamentation regulatory pathways also control changes in cell shape and polarity (Fig. S4A). The major changes can be genetically separated by examining mutants that are defective for a single aspect of invasive growth (adhesion, cell elongation, and cell polarity [119]). The elongated shape of filamentous cells occurs by an extension of the cell cycle (120–123), which results in increased apical growth through the polarisome complex (34, 119, 124–126). The polarisome complex includes the formin Bni1p and adaptors Pea2p, Spa2p, and Bud6p (127, 128). Cells lacking the polarisome protein Pea2p failed to develop an elongated morphology and did not form aggregates (Fig. 5A; Fig. S5, *pea2*Δ) (127). Similarly, a mutant with a hyperelongated morphology (26), generated by loss of the checkpoint kinase Hsl7p (129–131), made better aggregates (Fig. 5A; Fig. S5, *hsl7*Δ). At 72 h, cells extracted from aggregates were more elongated than cells in regular filaments (Fig. 5B). The fact that changes in cell shape can have macroscopic effects on aggregate morphology is consistent with a report describing how cell shape can have macroscopic impacts on colonial patterning (132).

10.1128/mSphere.00702-18.5 FIG S5 PWA of adhesion (*flo11*Δ), budding (*bud8*Δ), and elongation (*pea2*Δ) mutants and controls. Download FIG S5, TIF file, 1.0 MB. Copyright © 2019 Chow et al. 2019 Chow et al. This content is distributed under the terms of the Creative Commons Attribution 4.0 International license.

Cells undergoing filamentous growth bud at the distal pole by utilization of the distal-pole landmark Bud8p (119, 133, 134). Cells lacking Bud8p failed to form aggregates (Fig. 5A; Fig. S5, *bud8*Δ), and cells that always budded distally made somewhat better aggregates (Fig. 5A; Fig. S5, *bud4*Δ). Moreover, cells in pseudohyphal aggregates, visualized by overproduction of Flo11p, had a polarized morphology that aligned with the major axis of the aggregate (Fig. 5C). This orientation was dependent on Bud8p and the fMAPK pathway (Fig. 5D). Cells may collectively align to shape the aggregate pattern; alternatively, cells may share alignment as a result of packing forces experienced in aggregates. Therefore, an elongated cell morphology and distal-unipolar budding are important determinants of aggregate invasive growth.

Two observations further support a role for cell shape and polarity in aggregate-based responses. One observation came from examining aggregate responses during pseudohyphal growth. At colony perimeters, pseudohyphal cells made contact with each other to form pseudohyphal aggregates (Fig. S6). Time-course experiments showed that the cells extended away from colony centers in groups (Fig. S6A). During this response, polarized cells grew in parallel bundles (Fig. S6B and C, arrows). These features were observed in Σ1278b (Fig. S6A) and natural isolates of yeast (Fig. S6B and C), which showed differences in the types of aggregate pseudohyphal responses displayed.

10.1128/mSphere.00702-18.6 FIG S6 Aggregate-based responses are regulated by cell density and alcohols. (A) Time course of pseudohyphal growth in Σ1278b diploid strain (PC334). Cells grown on SLAD agar medium. Plates incubated at 30°C and imaged at 32 h, 36 h, 39 h, and 42 h. (B) Pseudohyphal aggregates formed at the colony perimeter by wild isolates W27 and S1 on SLAD medium. Addition of ethanol (2%) and Trp-OH (500 µM) induced assembly of filaments into large pointed aggregates. (C) Closeups of W27 forming pseudohyphal filaments in SLAD medium. Bar, 10 μm. Download FIG S6, PDF file, 1.5 MB. Copyright © 2019 Chow et al. 2019 Chow et al. This content is distributed under the terms of the Creative Commons Attribution 4.0 International license.

The second observation came from examining the aggregation of filamentous cells in liquid culture. Certain yeast strains can form flocs (135) and biofilms/mats (136, 137) in liquid culture (Fig. 5E). We found that groups of filamentous cells aggregated in liquid culture when grown under nutrient-limiting conditions (Fig. S7A and B, compare Glu to Gal). This facilitated examination of aggregate formation. The formation of aggregates in liquid and on surfaces is likely to be different; however, mutants defective for cell elongation (*pea2*Δ) and distal-pole budding (*bud8*Δ) were defective for aggregate formation during invasive growth as shown above (Fig. 5A) and in liquid culture (Fig. 5F). Moreover, the formation of liquid aggregates was dependent on nutrient levels. Liquid aggregates transferred to nutrient-rich conditions produced daughter cells that were rounder and budded axially, which led to nonaggregating cells (Fig. S7A, at right). Together, the data emphasize the role that cell shape and polarity play in filamentous aggregate responses in yeast.

10.1128/mSphere.00702-18.7 FIG S7 Aggregation in liquid culture is dependent on nutrient levels. (A) Images of cells under two conditions showing morphogenetic responses (100×). Blue dot indicates cell adopting a round phenotype; red arrow indicates axial budding. Bar, 500 μm. (B) Wild-type cells were grown for 4 h in YEP-Gal medium. At *t* = 0 h, cells were transferred to YEP-Gal or YEPD medium for the indicated times. Measurements of aggregate size were determined at 2 h, 4 h, and 24 h. Error bars represent standard error of the mean between independent experiments. (C) PWA of wild-type cells grown in the indicated concentrations. Download FIG S7, PDF file, 0.3 MB. Copyright © 2019 Chow et al. 2019 Chow et al. This content is distributed under the terms of the Creative Commons Attribution 4.0 International license.

### Alcohols and secreted metabolic enzymes promote aggregate invasive growth.

**FIG 6:**
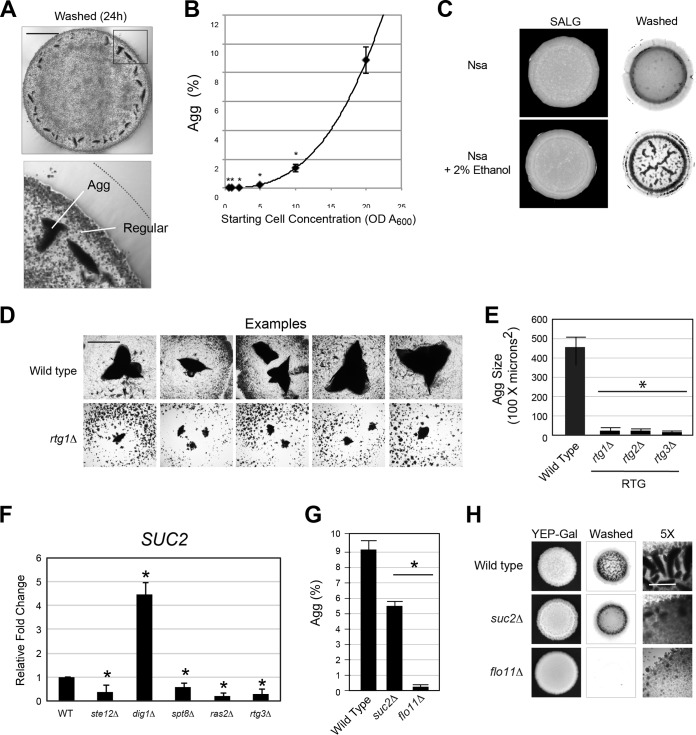
Cell density and secreted enzymes contribute to aggregate invasive growth. (A) One microliter of cells at an *A*_600_ OD of 10 was spotted onto YEP-Gal medium. Cells were incubated for 24 h at 30°C. The invasive pattern was photographed at 5×, and a composite image is shown. The line marks the colony perimeter at 24 h, prewash. White lines indicate aggregate and regular invasive growth. Bar, 1 mm. (B) Scatter plot of Agg (%), as a function of starting cell concentration. Asterisk, *P* value of <0.01, in reference to *A*_600_ of 20 on the *x* axis. Line shows the best fit, which resembles a nonlinear, cubic polynomial relationship (*y* = 0.0008*x*^3^ + 0.00064*x*^2^ − 0.0047*x*, *R*^2^ = 1). The equation was generated in Microsoft Excel. (C) Wild isolate NSA from a vineyard in Spain (181) grown on SALG (synthetic ammonium low-glucose medium [55]) with or without 2% ethanol as indicated. (D) Examples of aggregates formed on synthetic media for wild-type cells and the *rtg1*Δ mutant. Bar, 100 μm. (E) Average size of aggregates formed by the indicated mutants on S-Gal medium. Error bars represent standard error. Asterisk, *P* < 0.05. (Inset) Invasive pattern of wild-type cells and the *rtg3*Δ mutant. (F) Quantitative PCR analysis showing relative fold changes of *SUC2* in the indicated strains. Error bars represent standard deviation. (G) Aggregation [Agg (%)] of wild-type, *suc2*Δ, and *flo11*Δ cells. Asterisk, *P* value of <0.05. (H) PWA. Indicated strains were examined under standard aggregate-producing conditions. Far right, typical bright-field images are shown (5×). Bar, 500 μm.

Density-dependent responses are indicative of a microbial response known as quorum sensing (80, 138). As mentioned above, aggregates formed at colony perimeters where cells settle at high density (Fig. 6A). Cells settle more densely at colony perimeters due to a physical phenomenon known as the coffee-ring effect (139). Cells spotted at higher initial densities also made better aggregates (Fig. 6B; Fig. S7C). In microbes, soluble metabolic products can function as indicators of cell density (140, 141). In yeast, alcohols are waste products of metabolic activities, which can induce invasive growth and other responses (79, 80, 142, 143). Alcohols stimulated aggregate invasive growth (Fig. 6C) and aggregate pseudohyphal growth (Fig. S6B and C). The RTG pathway mediates ethanol-dependent invasive growth (55) and was also required for aggregate invasive growth (Fig. 6D and E). Although it is clear that different alcohols are sensed by different signaling pathways and in different ways, like tryptophol being recognized by the Tpk2p pathway (80), our results establish a link between alcohol and aggregate responses in yeast.

In microbial populations, secreted enzymes act as shared goods whose enzymatic activity produces products that are accessible to other cells in the population (83, 144–147). One of the major targets of the filamentation network (fMAPK, RAS, RTG, and SAGA) was the gene that encodes the secreted enzyme invertase or Suc2p (Chow et al., submitted). Invertase converts sucrose outside the cell into glucose and fructose, which can be transported into the cell. We confirmed that the expression of the *SUC2* gene was controlled by several key regulators of the filamentation signaling network, including fMAPK (*ste12*Δ and *dig1*Δ, Fig. 6F), SAGA (*spt8*Δ), RAS (*ras2*Δ), and RTG (*rtg3*Δ). Additionally, Suc2p has been shown to function as a shared good in S. cerevisiae (82, 144). Therefore, we tested whether Suc2p might influence aggregate invasive growth. Cells lacking Suc2p were defective for aggregate invasive growth, based on the size and number of aggregates (Fig. 6G and H). Suc2p might regulate aggregation as a shared metabolic enzyme or through functions outside its role as a glycolytic enzyme. Thus, diffusible alcohols and secreted metabolic enzymes promote aggregate invasive growth.

### fMAPK and RAS pathways are required for aggregate formation in directed selection experiments in the laboratory.

**FIG 7:**
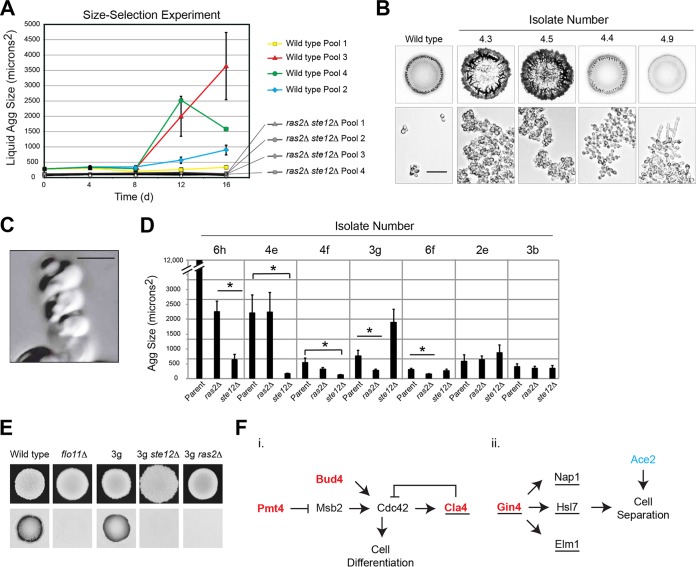
Directed selection experiments to define the role of signaling pathways and identify new regulators of aggregate formation in S. cerevisiae. (A) Aggregate size in liquid at the indicated times in a directed selection experiment. Four pools of wild type and four pools of *ras2*Δ *ste12*Δ mutant were size selected at the indicated times. (B) Top, PWA of the indicated evolved isolates, grown on YEP-Gal medium for 48 h. Bottom, bright-field microscopy (20×) of strains grown for 24 h in YEPD medium. Bar, 20 μm. (C) A *flo11*Δ evolved strain 4e cell exhibiting spiral growth. Rendering of serial Z-stack images at 100×. Bar, 5 μm. (D) Aggregate size in liquid of evolved *flo11*Δ parent strains with *ste12*Δ and *ras2*Δ disruptions. (E) PWA. Wild-type strain, *flo11*Δ strain, and *flo11*Δ evolved strain 3g, including *ste12*Δ and *ras2*Δ disruptions. Top, colony; bottom, washed plate. (F) Two pathways that impact differentiated multicellularity. Red font, genes identified and verified in this study. Underlining, genes found to impact multicellular phenotypes when deleted; blue font, identified previously in a separate study (86).

Directed selection experiments in the laboratory can identify new phenotypes and provide insights into biological functions. We performed directed selection experiments to characterize aggregate-based phenotypes that were generated in the laboratory. A size-selection experiment, similar to that reported in reference 83, was carried out for 20 days in liquid culture. Three out of the four pools produced aggregates after 16 days (Fig. 7A, wild-type, pools 1 to 4). To test whether the RAS and fMAPK pathways were required for the production of the liquid aggregate phenotypes, the same experiment was carried out with a mutant that lacked the fMAPK and RAS pathways. This strain failed to produce aggregates in liquid culture (Fig. 7A, *ste12*Δ *ras2*Δ, pools 1 to 4). Therefore, the fMAPK pathway and RAS pathways were required for the production of aggregate phenotypes in an unbiased directed-selection experiment.

To examine the phenotypes produced by the directed selection experiment, 15 isolates from the wild-type pools were examined. Isolates were examined by bright-field microscopy, and invasive growth was examined by the PWA. Different phenotypes were observed. A common pattern was that some isolates exhibited a more robust pattern of aggregate invasive growth, which corresponded to a “clumpy” cell phenotype (Fig. 7B, 4.3 and 4.5). A less common pattern (seen in one isolate) was a constitutive distal-pole budding pattern (Fig. 7B, 4.4). Yet another isolate showed heterogeneous cell morphologies populated with a high percentage of highly polarized cells (Fig. 7B, 4.9). The latter isolates did not show a dramatic increase in aggregate invasive growth. Therefore, different phenotypes led to different aggregate-based responses in liquid culture and on surfaces.

The clumpy cell phenotype was an expected outcome of the experiment, because increased cell adhesion might be expected to produce “multicellular” phenotypes (86). However, changes in cell shape and polarity represent potential new avenues toward achieving aggregation. To enrich for aggregates that arise due to morphogenetic changes, a strain lacking Flo11p was used as the parent strain (*flo11*Δ) in a separate size-selection experiment. In this experiment, most isolates showed heterogeneous cell morphologies (Fig. S8A). Among the phenotypes seen in colony-purified isolates were cells that formed spiral patterns (strain 4e, Fig. 7C), which is an unusual morphology for yeast yet one that is present in distantly related fungal species (148). To determine the roles of signaling pathways in generating these phenotypes, regulators of the fMAPK pathway or RAS pathway were disrupted in seven colony-purified isolates. In contrast to the size-selection experiment discussed above, most isolates showed some phenotypes that were independent of the RAS and/or fMAPK pathways. Specifically, isolates 4e and 3g retained polarized morphologies in the *ras2*Δ mutant, and isolates 4f, 3g, 6h, and 3b retained polarized morphologies in the *ste12*Δ mutant (Fig. S8). However, several isolates showed some dependency on either the fMAPK pathway, the RAS pathway, or both when analyzed for aggregate size (Fig. 7D) and cell morphology (Fig. S8B). One isolate was identified that restored invasive growth to the *flo11*Δ parent (isolate 3g, Fig. 7E). The fMAPK pathway was required for invasive growth of this isolate (Fig. 7E).

10.1128/mSphere.00702-18.8 FIG S8 Analysis of phenotypes derived from isolates obtained by directed selection experiments. (A) Microscopy (100×) of the indicated *flo11*Δ isolates derived from the directed selection experiment and their *ste12*Δ and *ras2*Δ derivatives. Typical examples are shown. Bar, 30 μm. (B) Dot plot showing cell lengths from isolates derived from directed selection applied to a *flo11*Δ parent. Isolates were also mutated to delete *STE12* and *RAS2*. Cell length was measured along a cell’s major axis. Values of individual cells are shown. Measurements were taken over several trials. Download FIG S8, PDF file, 0.7 MB. Copyright © 2019 Chow et al. 2019 Chow et al. This content is distributed under the terms of the Creative Commons Attribution 4.0 International license.

To identify mutations responsible for the phenotypes observed, the genomes of several isolates were analyzed by whole-genome sequencing. Raw genome sequencing data are available at the Sequence Read Archive (accession number PRJNA503202). Whole-genome sequencing identified several classes of genes that impacted aggregate phenotypes. We identified a single nucleotide (missense) mutation in one isolate (4.9) that would be expected to alter one amino acid of the Gin4p protein (G126V), the gene for which encodes a protein that promotes cell separation during cytokinesis (149). Another isolate (6h) contained multiple missense mutations that would be expected to impact the activity of the fMAPK pathway. One nonsense mutation that produced a premature stop codon at amino acid 386 was found in the *PMT4* gene, which encodes a glycosyltransferase that modifies Msb2p (150), the mucin-type glycoprotein at the head of the fMAPK pathway (25). Loss of Pmt4p results in underglycosylation of Msb2p and hyperactivation of the fMAPK pathway (29, 150). The other nonsense mutation produced a premature stop codon at amino acid 212 of the Cla4p protein, the gene for which encodes a p21-activated kinase and direct effector of Cdc42p (151) whose loss results in hyperpolarized growth (152). A third isolate contained a nonsense mutation at amino acid 565 of Bud4p, a mark for axial budding (153), which can account for the distal budding pattern seen in that isolate (isolate 4.4). These genes can be represented on a figure that involves Cdc42p-dependent pathways (Fig. 7F, i) and cell separation pathways (Fig. 7F, ii). One class that impacted cell separation, which we did not identify, has previously been described (Ace2p) (86). Therefore, multiple independent pathways can lead to aggregate phenotypes through mechanisms that involve morphogenetic pathway activity (RAS and fMAPK), changes to Cdc42p-dependent polarity, and cell separation.

## DISCUSSION

By examining filamentous growth in S. cerevisiae and C. albicans, we report here aspects of filamentous growth that involve the collective action of groups of cells in aggregates. Aggregate invasive growth involved the production of macroscopic divots by the surface penetration of large groups of cells. Signaling pathways that control filamentous growth were required for aggregate formation, including fMAPK, RAS, RIM101, RTG, SNF1, and SAGA. Given that the major signaling pathways that regulate filamentous growth also regulated aggregate invasive growth, we suggest that aggregate invasion is an extension of the normal filamentous growth response.

Our findings connect aggregate filamentous growth to a growing number of fungal colonial responses, including regular filamentous growth (20), biofilm/mat formation (37), and complex colony morphology (94, 103). The picture that is emerging from these studies is the ability of microbes to organize into highly complex structures (21, 154). Yeast can also undergo pseudohyphal growth in aggregates and form filamentous aggregates in liquid. These likely represent somewhat different responses, which may be regulated in different ways. Although the benefit of the responses is not yet clear, it seems that an obvious benefit to the formation of invasive aggregates is the increased penetration into surfaces, which may allow cells access to nutrient pools and new environments. Aggregates might result from amplified activity of the filamentation signaling network. More network activity would be expected to lead to more Flo11p protein, which might cause outward, growing cells to interact and bundle into deeply penetrating aggregates. We also show a connection between colony/mat pattern formation and aggregate invasive growth. Generally speaking, the function of colony pattern formation is not clear. In a related study, we show that cell adhesion in colonies protects cells from predation from grazing by macroscopic predators and also functions to promote heat dissipation (Chow et al., submitted). Here, we show that colony/mat ruffling contributes to the formation of invasive aggregates.

Signaling pathways control aggregate invasive growth through mechanisms that are known to influence cooperation among cells in yeast and other microbial species (155–157). Microbial systems, like the Gram-negative bacterium *Myxobacterium* (158), slime mold *Dictyostelium* (159), and budding yeast Saccharomyces cerevisiae (160, 161), have emerged as useful models for understanding the genetic basis of cooperation. Through homotypic interactions, cell adhesion molecules can promote interactions among like individuals (135, 162, 163), and we show here that the major cell adhesion molecule Flo11p is required for aggregate invasive growth. Secreted metabolic enzymes produce products that are accessible to neighboring individuals in the community, which is known to favor cooperation among individuals (82, 83, 164). Aggregate invasive growth was induced by the secreted enzyme invertase. Aggregate invasive growth was also stimulated by alcohols, which are thought to serve as indicators of cell density in yeast and other species (80, 165). Therefore, cooperation in terms of cell adhesion, sharing metabolites, and communication about cell density all appear to impact aggregate formation.

During filamentous growth, mother and daughter cells remain attached in a response that can be thought of in cooperative terms as “staying together” (166). During aggregate invasive growth, groups of filaments “come together” in a type of quasisocial response (166). In this way, filamentous growth may involve behaviors where individuals become elongated and grow outward to reach out and make adhesive contacts with neighboring filaments. This response presumably occurs in the wild. Yeast form aggregates under normal growth conditions in strains (Σ1278b background and natural isolates) that have retained traits that were lost in other laboratory strains (78, 167). Aggregate invasive growth is also reminiscent of colonization patterns of S. cerevisiae on plant surfaces (168). The aggregate response also occurs in the major human pathogen C. albicans, which resembles patterns seen in C. albicans infections (169, 170). Therefore, the response characterized here might be widespread among different species and occur in diverse environments. Aggregate behaviors are likely to impact the overall understanding of fungal foraging responses, including aspects of fungal pathogenesis.

Recently, directed selection approaches have identified new regulatory aspects of cellular interactions (84–87). Here, a directed selection experiment was used to identify regulators of aggregate responses in S. cerevisiae. By this method, we identified several regulators of aggregate behaviors, which include expected regulators and effectors of morphogenetic pathways, genes that control Cdc42p-dependent bud-site selection, and genes that control cell separation. A connection between bud-site selection and cell separation has been previously established (171). These results reinforce the idea that filamentous growth pathways are critical for aggregate behaviors and suggest that diverse paths can lead to collective responses in fungi.

## MATERIALS AND METHODS

### Strains and plasmids.

Strains are listed in Table S1 in the supplemental material. Yeast and bacterial strains were grown by standard methods (172, 173). Gene disruptions and *GAL1* promoter fusions were made by PCR-based methods (174, 175), including antibiotic-resistant markers (176) and epitope fusions (177). Integrations were confirmed by PCR analysis and phenotype. Strains expressing green (GFP) and red fluorescent proteins (mCherry) were generated as follows. A plasmid containing *pTEF2-mCherry-URA* (JW1300 [178], provided by the Weissman Lab [UCSF]) was used as a template to amplify *pTEF2-mCherry-URA* by PCR. Integration of the *pTEF2-mCherry-URA* cassette at the *ura3-52* locus in a wild-type strain (PC6021) was used to construct strain PC6581. A GFP-labeled strain (PC6733) was generated by insertion of the *NAT* cassette (PC2205) (176) to replace the *HIS5* gene in strain PC6581 to make PC6735, followed by integration of *GFP*γ*-HIS* (179) to replace mCherry. GFPγ-HIS was obtained from Addgene (catalog no. 44859). A plasmid containing GFP-2×PH (PC2560, CS189 [180]), provided by the Emr Lab (Cornell University), was used for some experiments. Nsa (181) and S1 (182) strains are natural S. cerevisiae isolates from vineyards. W27 (183) is a commercially available yeast strain.

10.1128/mSphere.00702-18.9 TABLE S1 Yeast strains used in the study. Download Table S1, XLSX file, 0.03 MB. Copyright © 2019 Chow et al. 2019 Chow et al. This content is distributed under the terms of the Creative Commons Attribution 4.0 International license.

10.1128/mSphere.00702-18.10 TABLE S2 Screen of C. albicans knockout library for altered aggregate formation. Download Table S2, XLSX file, 0.2 MB. Copyright © 2019 Chow et al. 2019 Chow et al. This content is distributed under the terms of the Creative Commons Attribution 4.0 International license.

### Invasive aggregate formation, enrichment, and analysis.

The plate-washing assay (PWA) was performed as described previously (23). Standard aggregate-inducing conditions were as follows. Semisolid agar medium was prepared by dispensing 25 ml medium per plate. Plates were stored for 3 days at 25°C and wrapped to maintain a consistent moisture level. Cells were grown at 30°C for 16 h in liquid YEPD medium. Approximately 10^7^ cells were washed twice in distilled water and resuspended in water to a calculated OD *A*_600_ of 20. To evaluate aggregate formation, the PWA was performed with 10 μl of OD *A*_600_ = 20 cells on YEP-Gal for 48 h unless otherwise described. Aggregates formed under specific conditions; they formed different shapes under different conditions and in different species. All comparisons were made using the same parent strain and the same conditions.

To separate populations of cells invading as individual filaments or aggregates, wild-type cells were spotted on YEP-Gal plates at a high concentration (*A*_600_ = 20), to enrich for aggregates, and a low concentration (*A*_600_ = 0.2), which resulted in uniform invasion of individual filaments. Plates were incubated for 72 h at 30°C. Plates were washed, and invaded cells were excavated with toothpicks and placed in centrifuge tubes containing 200 μl of water. Large pieces of agar were broken up with a pipette tip to further separate invaded cells from the agar. In some instances, further separation required melting the agar at 90°C for 5 min. Heated tubes were spun for 30 s at 15,000 rpm to separate melted agar from pelleted cells.

To evaluate the impact of environmental stimuli on aggregate formation, cells were grown under the following conditions: YEPD + 8% Glu for high nutrient, YEP-Gal + 1 M KCl for high osmolarity, and agar medium that was air dried for 4 to 5 days for humidity. To evaluate the role of pH, YEP-Gal plates were treated by spotting 5 μl 2 N HCl or 2 N NaOH and then aliquoting cells over the dried spots. For some experiments, phenol red (184) was added to YEPD at a concentration of 20 mg/liter to measure the pH of the medium. Synthetic ammonium low glucose (SALG) was prepared as described previously (55).

Quantitation of aggregate invasion was performed as follows. Photographs of colonies and invasive scars were taken using an Evolution MP color camera (Media Cybernetics) and Q Capture software. Images were imported into ImageJ and converted to 8-bit for analysis. The Threshold tool was used to select regions of the image to measure. To determine the colony surface area, images were set a threshold that was sufficient to exclude the area surrounding the colony and quantitated using the Analyze Particles tool. The area of aggregates was determined by setting the threshold to a value that was sufficient to include aggregates but exclude the invasive growth of individual filaments. Aggregates were then expressed as a percentage of colony surface area. Values were the average for at least three separate colonies. Error bars show the standard error. All statistics were performed using an unpaired Student *t* test.

The depth of agar invasion was determined by microscopy by measuring the distance between the first cell passing through the focal plane of the microscope and the last cell exiting the focal plane of the microscope. Best-fit models and *R*^2^ values were obtained using Excel. To determine the length-to-width ratio of cells, the long and short axes of cells were measured by ImageJ. Samples from at least 3 independent experiments were compared, and the average values were shown. ImageJ was also used to quantitate fluorescent intensity of cells expressing Flo11-HA (http://www.di.uq.edu.au/sparqimagejblots). Quantitation of band intensity was determined by ImageJ. Immunofluorescence images were imported into ImageJ as 8-bit JPEG images. The ImageJ thresholding tool, which can determine the brightest part of an image, was used to determine signal strength and localization.

To determine the alignment of cells in an aggregate, electron micrographs were imported into ImageJ. The aggregate axis was estimated by eye, and the photo was reoriented to set the aggregate axis to zero. The line tool was used to draw a line from the leftmost side of a cell, along its length, to the rightmost side of a cell to ensure an angle measurement from +90° to −90° relative to the aggregate axis. A data set was created for each strain where cells that fell within +20° to −20° relative to the aggregate axis were assigned a value of 1 and cells outside that range were assigned a value of 0. To determine whether or not the alignment was random, data sets were compared to three simulated random data sets of comparable size created by the random number generator function in Excel.

To determine size of liquid aggregates, cells were grown for 16 h in YEPD medium and transferred to YEP-Gal medium at *t* = 0. Samples were harvested at 1-h intervals, and photographs ertr taken with an Axioplan 2 fluorescence microscope (Zeiss) with a 20× objective. Images were imported into ImageJ where liquid aggregates were identified by the Find Edge tool and size was determined by the Threshold tool.

### Pseudohyphal aggregate formation assays.

Diploid strains were grown for 16 h in SD + AA medium at 30°C. Cells were washed with dH_2_O and spotted onto SLAD medium [1× YNB, 2% glucose, 50 μM (NH_4_)_2_SO_4_, and 2% agar (22)]. Plates were incubated at 30°C. Peripheral morphology was observed by microscopy. For some experiments 2% ethanol or Trp-OH (500 μM) was added as a supplement to agar plates.

### Scanning electron microscopy.

Scanning electron microscopy (SEM) was performed as described previously (185). Cells were grown for 16 h in liquid YEPD medium. Approximately 2 μl of cells with an *A*_600_ of 0.02 was spotted onto 35-μm microsieves (BioDesign Inc. of New York; catalog no. N35S), set onto semisolid YEP-Gal (2% agar) medium, and allowed to grow for 24 h. Sieves were transferred to petri dishes for fixation (2% glutaraldehyde for 4 h at 4°C) and dehydration (30%, 50%, 70%, 80%, 85%, 90%, and 95% ethanol for 15 min) and washed twice in 100% ethanol for 15 min. Samples were critical point dried in 50% hexamethyldisilazane (HMDS) and 50% ethanol for 1 h before the final critical point drying step in 100% HMDS for 16 h. Samples were carbon coated and imaged on a Hitachi S4000 field emission scanning electron microscope (FESEM).

### Fluorescence microscopy.

Wild-type cells with integrated pTEF2-mCherry (PC6581) and pTEF2-GFPγ (PC6733) were grown for 16 h in liquid YEPD, concentrated to an *A*_600_ of 20, and mixed in a 1:1 ratio. Ten microliters of cells was spotted onto YEP-Gal (2% agar) medium and incubated for various time periods. Plates were washed and examined by differential interference contrast (DIC) and fluorescence microscopy using an Axioplan 2 fluorescence microscope (Zeiss) with 5×, 10×, 20×, 40×, and 100× Plan-Apochromat 100×/1.4 (oil) (numerical aperture [NA], 0.17) objectives. Digital images were obtained with the Axiocam MRm camera (Zeiss). Axiovision 4.4 software (Zeiss) was used for image acquisition. Digital images were imported into ImageJ (https://imagej.nih.gov/ij/) in 8-bit format. Brightness and contrast were adjusted to reduce background. The threshold function was used to estimate the percentage of mCherry- or GFPγ-expressing cells in an aggregate. In total, 10 aggregates were analyzed from three independent trials.

To observe multicellular interactions in liquid, wild-type cells expressing mCherry and wild-type cells expressing GFPγ were grown separately to stationary phase in liquid YEPD medium. Two hundred microliters of each culture was washed twice in distilled water and transferred to 5 ml of YEPD or YEP-Gal. Samples were photographed by differential interference contrast (DIC), bright-field, and fluorescence microscopy. Serial Z-stack images showing contact between pTEF2-mCherry- and pTEF2-GFPγ-expressing strains were examined with the reslice function in ImageJ to view cross sections of potential points of contact.

### C. albicans deletion collection screen.

C. albicans deletions constructed by Noble et al. (107), Mitchell and colleagues (108–112), and Homann et al. (113) were obtained from the FGSC (http://www.fgsc.net/candida/FGSCcandidaresources.htm). Pilot experiments were performed to determine the optimal conditions for aggregate invasion of C. albicans (48 h at 37°C on YEPD + URA semisolid medium). Approximately 1,186 gene deletion knockouts, representing approximately 850 unique gene deletions and 5 wild-type controls, were pinned onto agar medium in Omnitrays. Plates were photographed at different angles (to account for glare effects), washed in a stream of water, and photographed again. Colony centers were ignored due to pinning artifacts. Invasive scars were visually scored for regular invasive growth and aggregate invasion for each mutant. Colonies were compared to wild-type controls and neighboring colonies to identify hits, which were classified as “strong” or “weak” based on phenotype.

The primary screen identified 132 unique hits. Bioinformatics and GO term analysis identified 80 mutants that would be expected to regulate aggregate formation. These included regulators of the Cek1p MAPK pathway (*MSB2*, *CST20*, *CPP1*, and *CPH2*), which is the major MAPK pathway that regulates filamentous growth in C. albicans. Components of the HOG pathway (*SSK2*, *PBS2*, and *HOG1*) and the Rim101 pathway (*VPS28*, *SNF7*, *RIM9*, *RIM13*, *RIM101*, and *NRG1*) were also uncovered. Genes involved in adhesion (*RBT1*, *HYR4*, and *YWP1*), protein glycosylation/folding (*KRE5*, *MNN14*, *MNN9*, and *OCH1*), nutrition (*LIP4*, *OSH3*, *COX4*, *DAC1*, *FAD3*, and *TSC11*), and the cell cycle (*CCN1*, *HSL1*, *CDC10*, and *CLB4*) were identified. Genes were also uncovered that, although not explicitly shown to impact aggregate formation, have been implicated in biofilm formation, hyphal growth, and virulence and would be expected to impact the formation of aggregates; these include genes related to the cell wall (*ORF19.3010.1*, *HYR1*, *ORF19.6741*, and *ORF19.12732*), chromatin remodeling (*SNT1*, *SET3*, *BCR1*, *ISW2*, *ORF19.10953*, and *ORF19.4729*), Ca^2+^ ion regulation (*SPF1* and *MID1*), lipid modification (*INP5*, *STT4*, and *SLD1*), protein modification (*SIT4*), replication (*DPB4* and *HFL1*), signaling (*PDE2*, *GPA2*, *KIC1*, *DFG5*, *RHO3*, *RHB1*, *CMP1*, *GIN4*, and *LRG1*), sporulation (*ORF19.5644*), trafficking (*YPT72*, *APM1*, *KAR3*, and *PEP7*), transcription (*RFG1*, *PHO4*, *RAP1*, *NDT80*, *DAL81*, *SFL2*, *BRG1*, *CAS5*, *TYE7*, *ROB1*, *RBF1*, *RCA1*, *ACE2*, *SSN6*, *AHR1*, *HMS1*, and *ORF19.1577*), and the vacuole (*RAV2*). In addition, we identified 6 genes of unknown function, including 2 with some role in filamentous growth (*ORF19.6874* and *ORF19.267*), 1 with a role in biofilm formation (*ORF19.5412*), and 3 without any defined role (*ORF19.194*, *ORF19.2200*, and *ORF19.6449*).

We probed 8 unique genes by a secondary screen. The genes and the *S. cerevisae* strain(s) used (in parentheses and brackets) were as follows: *PEX8* (*pex3*Δ [PC3097]), *ALG3* (*alg8*Δ [PC5396], *alg9*Δ [PC5399]), *SPT8* (*spt8*Δ [PC4008]), *ORF19.5300* (*cne1*Δ [PC5926]), *ORF19.5326* (*mig2*Δ [PC5058]), *SWI4* (*swi4*Δ [PC3428]), *MNN10* (*mnn10*Δ [PC5400]), and *ECM14* (*ecm14*Δ [PC3413]). From this analysis, 7 new unique regulators of aggregate invasion in S. cerevisiae were uncovered, classified according to the following processes: transcription (*SPT8* and *MIG2*), protein glycosylation/folding (*ALG8*, *ALG9*, *CNE1*, and *MNN10*), and the peroxisome (*PEX3*).

### Evaluation of SUC2 expression levels.

To compare the transcriptional response of wild-type (PC538) and the *ste12*Δ (PC1079)*, dig1*Δ (PC3039)*, rtg3*Δ (PC3642), *spt8*Δ (PC4008), and *ras2*Δ (PC562) mutants, cells were concentrated (OD *A*_600_ = 2-0) and spotted in 10-μl aliquots onto YEP-Gal (2% agar) for 24 h. Cells were spotted in six colonies per plate equidistant to each other and the plate center. All six colonies were harvested for each trial, and three separate trials were compared for each strain. The entire colony surface was scraped into 500 μl of distilled water, harvested by centrifugation, washed, and stored at −80°C. RNA was harvested by hot acid phenol-chloroform extraction as described previously (186). Samples were further purified using the Qiagen RNeasy minikit (catalog no. 74104; Qiagen, Hilden, Germany). RNA concentration and purity were measured using NanoDrop (NanoDrop 2000C; Thermo Fisher Scientific, Waltham, MA, USA). RNA stability was determined by running the sample on an agarose gel.

cDNA libraries from RNA samples were generated using iScript Reverse Transcriptase Supermix (Bio-Rad; catalog no. 1708840). qPCR was performed using iTaq Universal SYBR Green Supermix (Bio-Rad; catalog no. 1725120) on a Bio-Rad CFX384 real-time system. Fold changes in expression were determined by calculating ΔΔ*C_T_* using *ACT1* mRNA as the housekeeping gene for each sample. Experiments were performed with biological replicates, and the averages for multiple independent experiments were recorded.

### Immunofluorescence analysis.

Immunofluorescence was performed as described previously (75). To determine the localization of Flo11-HA, cells enriched from invaded aggregates and nonaggregates were resuspended in 200 μl of water. To further enrich for aggregates, cells were grown under standard aggregate-inducing conditions, using YEP-Gal plates that had dried for 5 days, as desiccation produced better aggregates. Cells were fixed by adding 60 μl of 37% formaldehyde and incubated by on end-on-end rotation for 1 h at room temperature. Cells were resuspended in 200 μl fixative (0.74 ml dH_2_O, 22.5 μl 2 N NaOH, 260 μl 15% paraformaldehyde, 0.08 g KH_2_PO_4_) and incubated by end-on-end rotation for 16 h at room temperature. Cells were immobilized on polylysine-coated multiwell slides (MP Biomedicals; catalog no. 096041205) and allowed to settle for 15 min. Cells were washed 3 times in phosphate-buffered saline (PBS) containing 2% bovine serum albumin (BSA) and incubated in PBS containing 2% BSA for 1 h. Cells were stained with DyLight550 anti-HA antibodies (Thermo Fisher; catalog no. 26183-D550) resuspended in 2% BSA in PBS at a concentration of 1:100. For the tracking of Flo11p-HA localization, p*GAL-FLO11-HA* was grown for 16 h in YEPD, and approximately 1 ml of cells was harvested by centrifugation, washed in 2 volumes of distilled and deionized water, and transferred to 10 ml of YEP-Gal to induce expression of *FLO11-HA*. Cells were fixed as described above at *t* = 0 h, 0.5 h, 1 h, 2 h, and 4 h. Flo11p-HA was probed as described above. Imaging of Flo11p-HA filaments and aggregates was at the same exposure. Immunofluorescence signal was quantitated using gel analysis tools in ImageJ. Total signal was averaged per cell, 15 cells in normal filaments and 30 cells in aggregates.

Pulse experiments were performed by growing Flo11-HA and pGAL-Flo11-HA strains for 16 h in YEPD liquid medium. Cells were washed twice in dH_2_O, transferred to 10 ml liquid YEP-Gal medium to induce expression of *FLO11*, and grown for 4 h before fixing and imaging.

### Immunoblot analysis.

Immunoblot analysis was performed as described previously (29). Cells enriched from invaded aggregates and nonaggregates were boiled at 110°C for 5 min and centrifuged at 15,000 rpm for 30 s to separate cells from molten agar. Cells were resuspended in Thorner buffer (40 mM Tris, pH 8, 5% SDS, 8 M urea, 100 μM EDTA) and vortexed with glass beads for 10 min. Cell extracts were separated by sodium dodecyl sulfate-polyacrylamide gel electrophoresis (SDS-PAGE) (6% acrylamide) and transferred to nitrocellulose membranes. Membranes were probed with mouse anti-HA primary antibodies (Roche; catalog no. 11583816001) and mouse anti-Pgk1 (Novex; catalog no. 459250). Secondary antibodies were goat anti-mouse (Bio-Rad; catalog no. 170-6516). Blots were visualized by chemiluminescence using a Bio-Rad ChemiDoc XRS+. Ponceau S staining was used to confirm equal protein loading between samples.

### Comparative analysis of aggregate development in different strains.

Screening for aggregate phenotypes was based on previous studies (83–86, 187). Aggregate isolates were separated by size by centrifugation in 25% polyethylene glycol 3000 (PEG 3000). As a proof-of-principle experiment, a *flo11*^−^ *ADE*^+^ strain (PC1029) was grown in a mixed culture with a *FLO11*^+^ *ade*^−^ strain (PJ69-4A), which grows in clusters of 4 to 10 cells. One round of growth and selection by centrifugation enriched for the larger *FLO11*^+^ *ade*^−^ aggregates based on colony color.

Wild-type cells (PC538) and the *ste12*Δ *ras2*Δ (PC2511), *flo11*Δ (PC1029), *flo11*Δ *ras2*Δ (PC2507), and *flo11*Δ *ste12*Δ (PC2505) mutants were compared in a directed selection experiment for multicellular development. The *flo11*Δ (PC1029) mutant was used to eliminate selection for increased adhesive contacts (or “snowflakes” in reference 84). Cells from a purified single colony were grown for 16 h in 5 ml of YEPD at 30°C with shaking at 220 rpm (Barnstead Lab-Line; MaxQ 3000). Approximately 500 μl of cells from a saturated culture was resuspended atop a 25% PEG (Sigma-Aldrich, catalog no. P4338) solution (5 ml) in a 15-ml conical tube. Samples were centrifuged (International Equipment Co.; model CL) for 7 s at 13,000 rpm to separate cells by size. By round 10, wild-type cells formed large liquid aggregates and were centrifuged for less time (5 s on day 10, 1 s on day 15). After centrifugation, the top 4.5 ml was removed, the remaining 500 μl was transferred to 5 ml of fresh YEPD, and the culture was incubated for 16 h at 30°C. Cells were examined by microscopy (20×) to detect morphological changes and frozen in glycerol stocks at 4-day intervals. The experiment was ended at 20 rounds of selection (20 days, 100 generations).

Selected strains were colony purified and evaluated by PWA (for colony morphology, invasive growth, and aggregate formation) and microscopy to identify phenotypically unique isolates. Six isolates were selected for whole-genome sequencing: 3.1 and 4.3 showed enhanced aggregate invasion at colony perimeters, 3.4 and 4.5 showed enhanced aggregate invasion underneath the entire colony surface, 4.4 showed increased distal-pole budding, and 4.9 showed a hypha-like morphology by microscopy. To compare the sizes of multicellular aggregates, cells were grown to saturation in YEPD and examined by microscopy at 20×. Images were imported into ImageJ for analysis. A macro was written for batch processing (Liquid Aggregate Area.txt) to identify and estimate the size of liquid aggregates.

In a separate experiment to enrich for Flo11p-independent isolates, the *flo11*Δ (PC1029) mutant was mutagenized with ethyl methanesulfonate (EMS) to generate between 5% and 17.5% killing. A mock-treated sample was carried along as a control. Mutagenized cells were separated into 6 cultures and grown in 10 ml of YEPD liquid cultures for 16 h. From overnight cultures, 1 ml of cells in YEPD was applied to 10 ml of 25% PEG. After a quick centrifugation, the bottom 0.5 ml of cells was used to inoculate 10 ml fresh YEPD medium. The selection experiment was repeated for 15 rounds (75 generations). After four rounds of selection, cells became clumpier, so 300 μl of cells was washed and resuspended in water to reduce clumpiness. For the final selection, cells were incubated with vigorous shaking for 5 h, and the bottom 1 ml of cells was transferred after 5 min of settling time. Multiple lines were rescued from each of the 6 tubes and separated by phenotype based on scoring for morphological phenotypes. Ten lines were chosen for detailed analysis (2f, 3e, 3g, 3k, 4e, 4f, 5f, 6a, 6f, and 6h), which included microscopy, growth on SD-HIS with or without aminotriazole (Sigma-Aldrich; catalog no. A8056) to evaluate the activity of a *FUS1-HIS3* reporter (25), staining with calcofluor white, and genetic analysis by deletion of the *STE12* and *RAS2* genes.

### DNA sequencing analysis.

Isolates from the directed selection experiment (3.1, 3.4, 4.3, 4.4, 4.5, and 4.9) were analyzed by whole-genome sequencing. Isolate strains were grown for 16 h in liquid YEPD and pelleted. Genomes from isolates were extracted using the Qiagen Gentra Puregene yeast/bacterium kit (catalog no. 158567). DNA concentration and purity were measured using NanoDrop (NanoDrop 2000C). After generating variant calls using the GATK haplotype caller, instances where the wild type differed from other strains were identified. Using thresholds where either wild-type alternate (ALT) ≤ 25% and any mutant ALT ≥ 75% or wild-type ALT ≥ 75% and any mutant ALT ≤ 25% resulted in unique loci.

### Data availability.

Raw genome sequencing data are available at the Sequence Read Archive under accession no. PRJNA503202.
