# Supplementary figures and images for: Aggregate Filamentous Growth Responses in Yeast
Source: mSphere. 2019 Mar 6;4(2):e00702-18. doi: 10.1128/mSphere.00702-18 (PMC6403458; doi:10.1128/mSphere.00702-18)

A

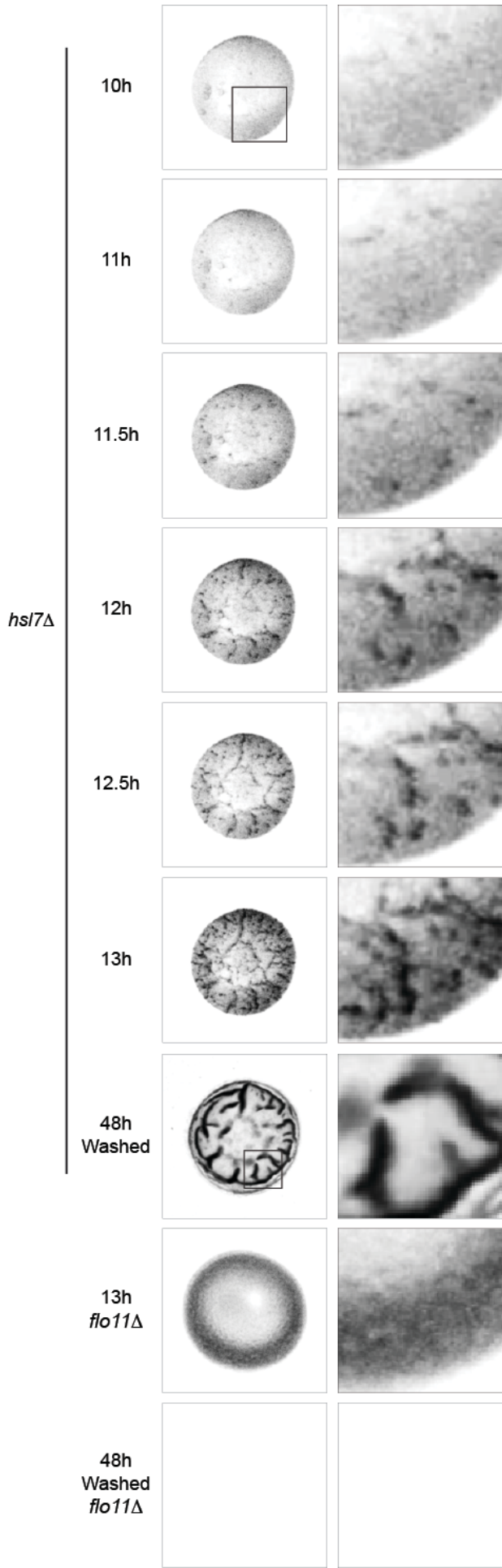

B

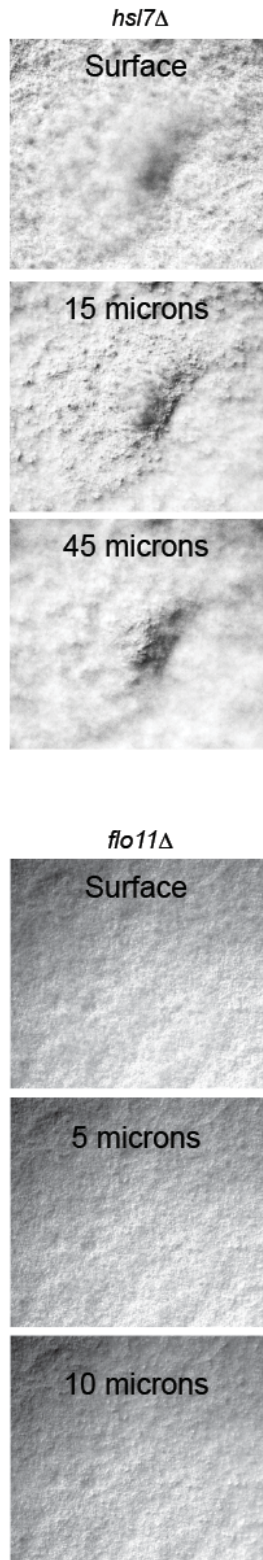

C

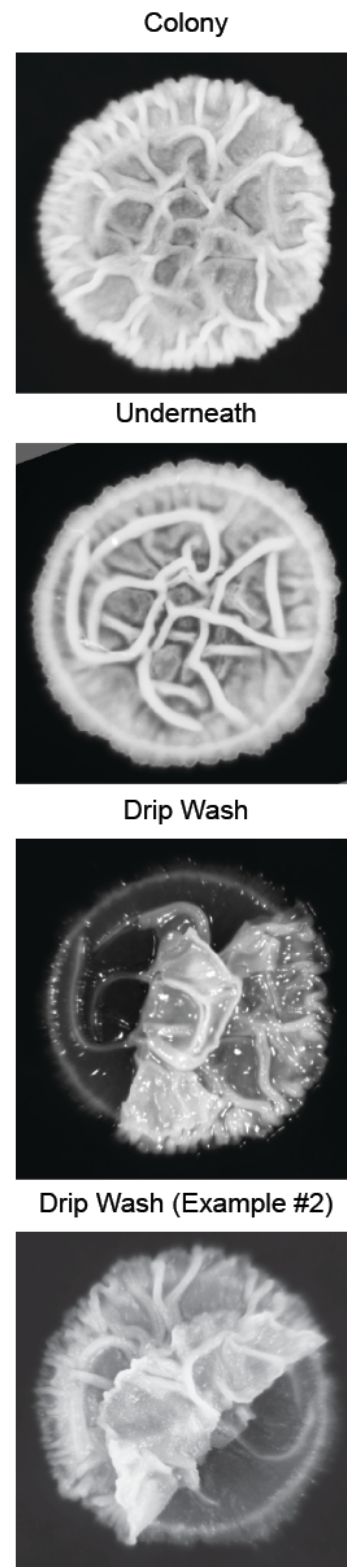

Supplement: FIG S1 [file mSphere.00702-18-sf001.pdf]

A

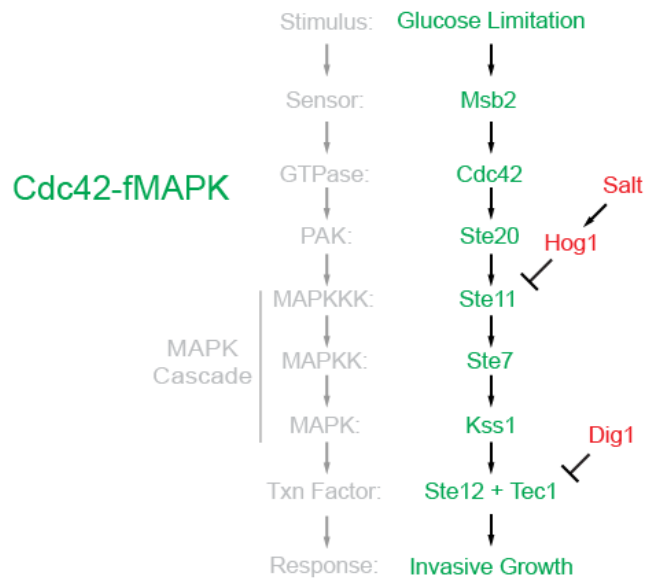

B

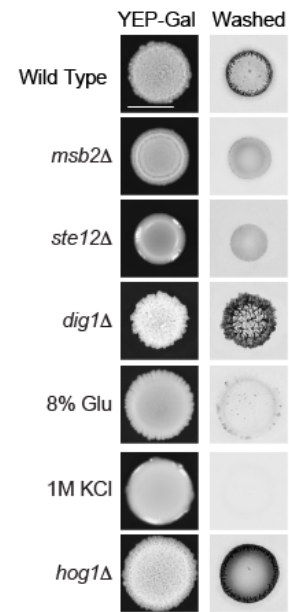

C

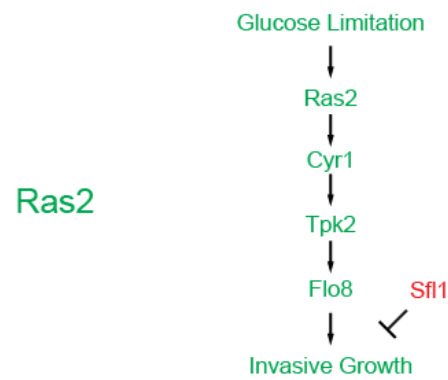

D

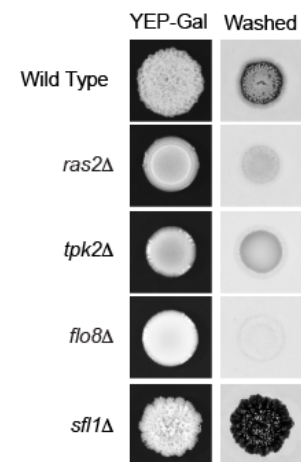

E

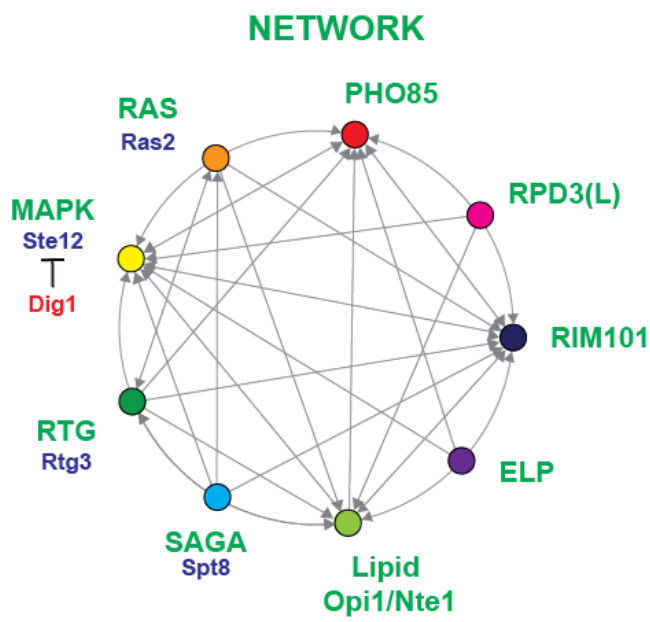

F

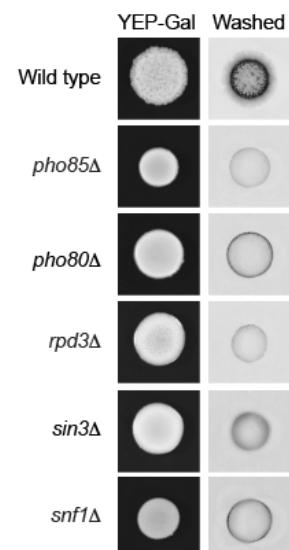

Supplement: FIG S2 [file mSphere.00702-18-sf002.pdf]

**Fig.\_S3**

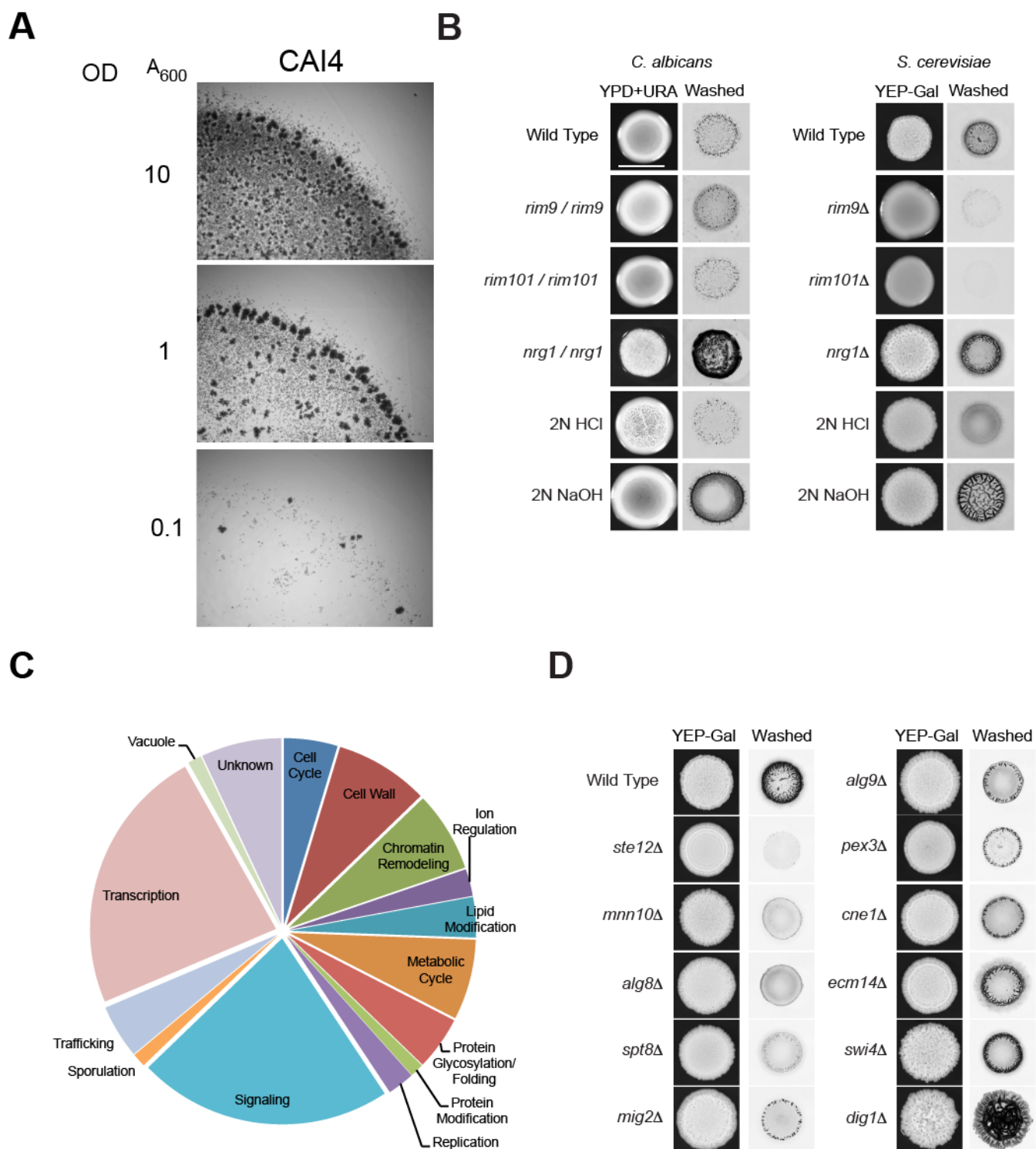

Supplement: FIG S3 [file mSphere.00702-18-sf003.pdf]

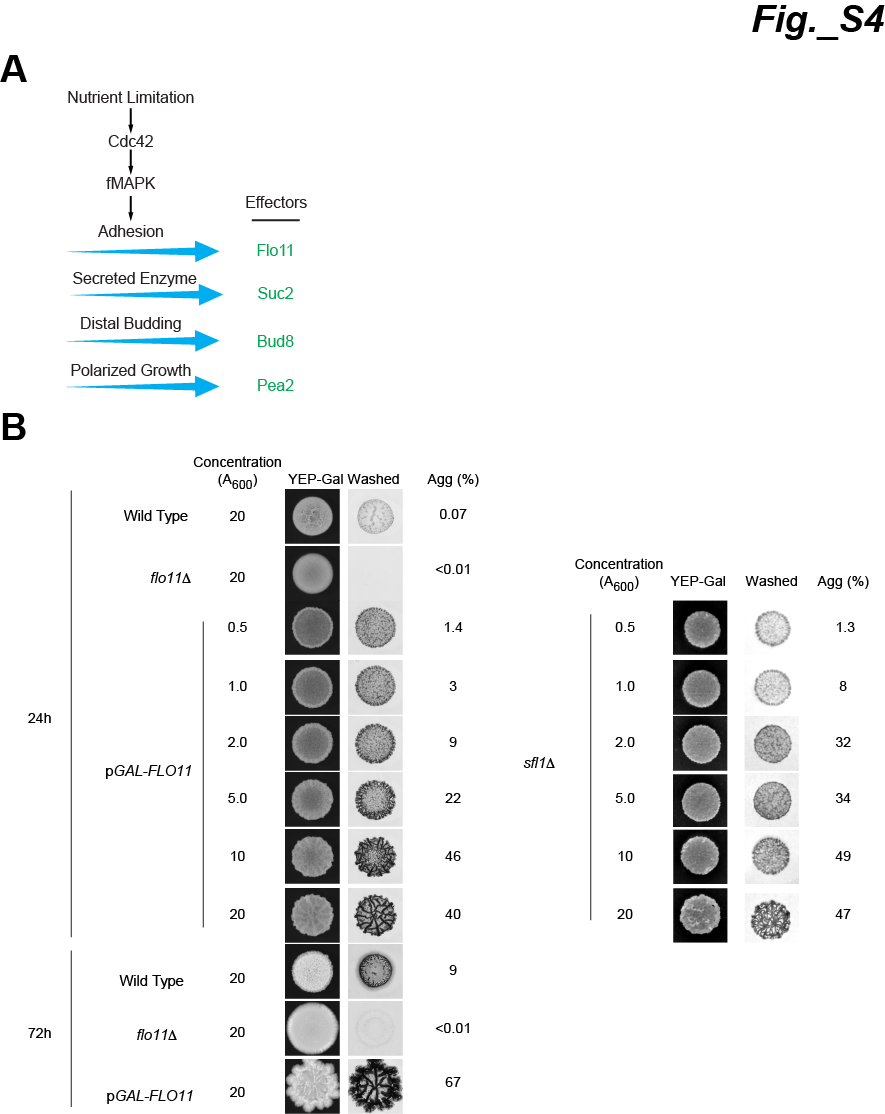

Supplement: FIG S4 [file mSphere.00702-18-sf004.tif]

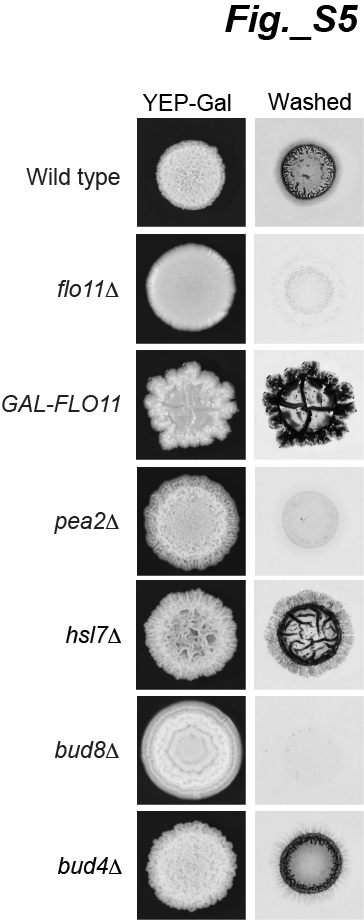

Supplement: FIG S5 [file mSphere.00702-18-sf005.tif]

**A**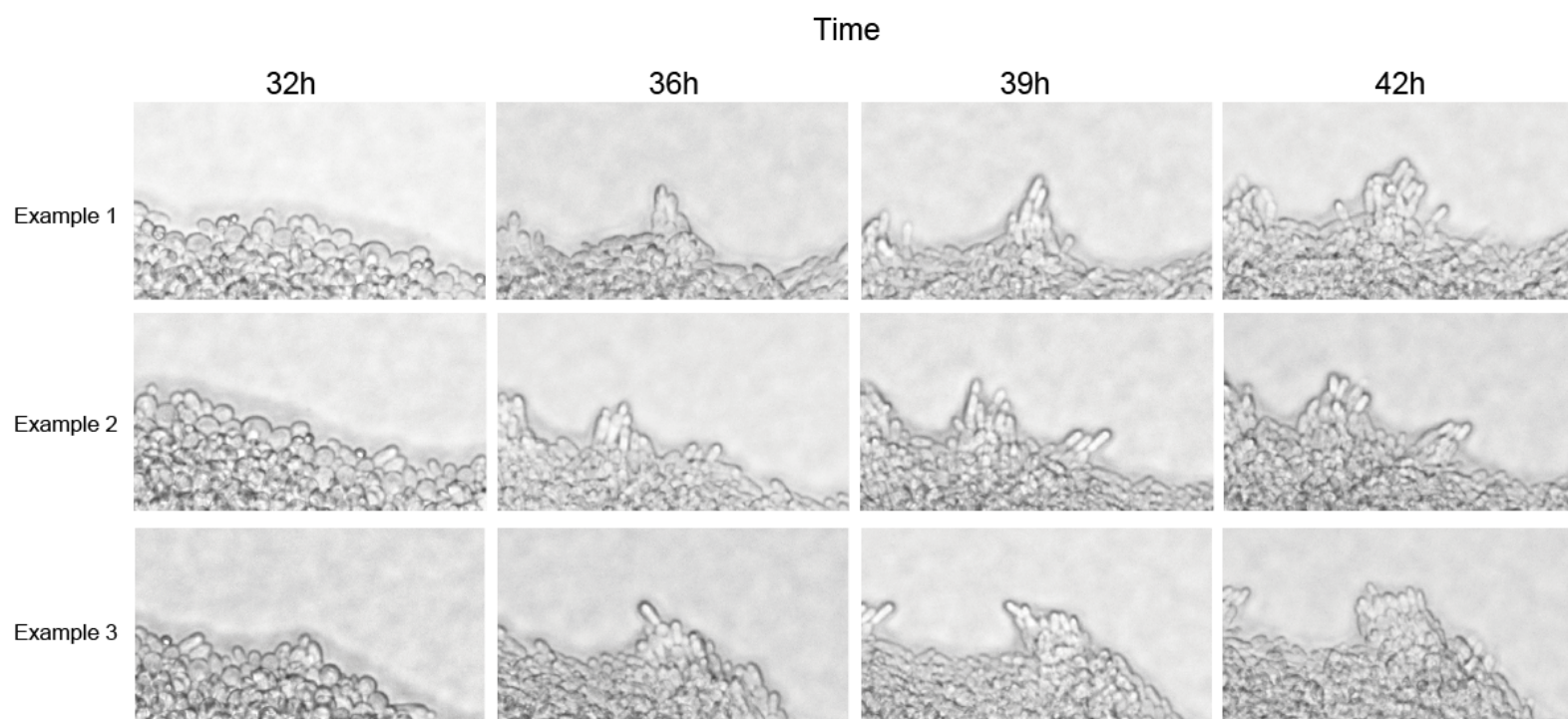**B**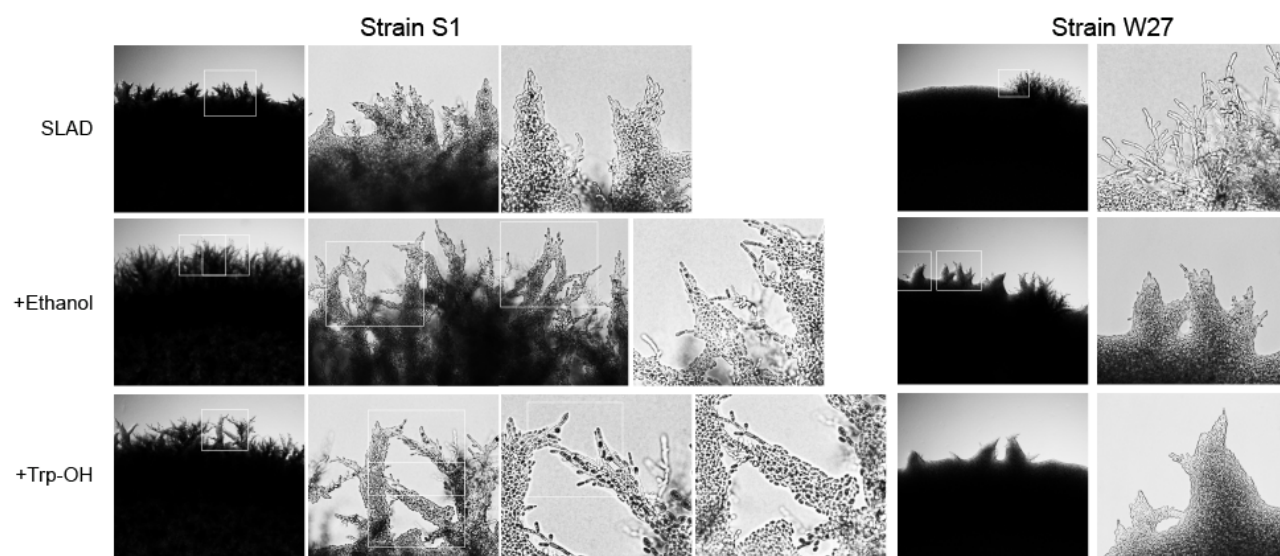**C**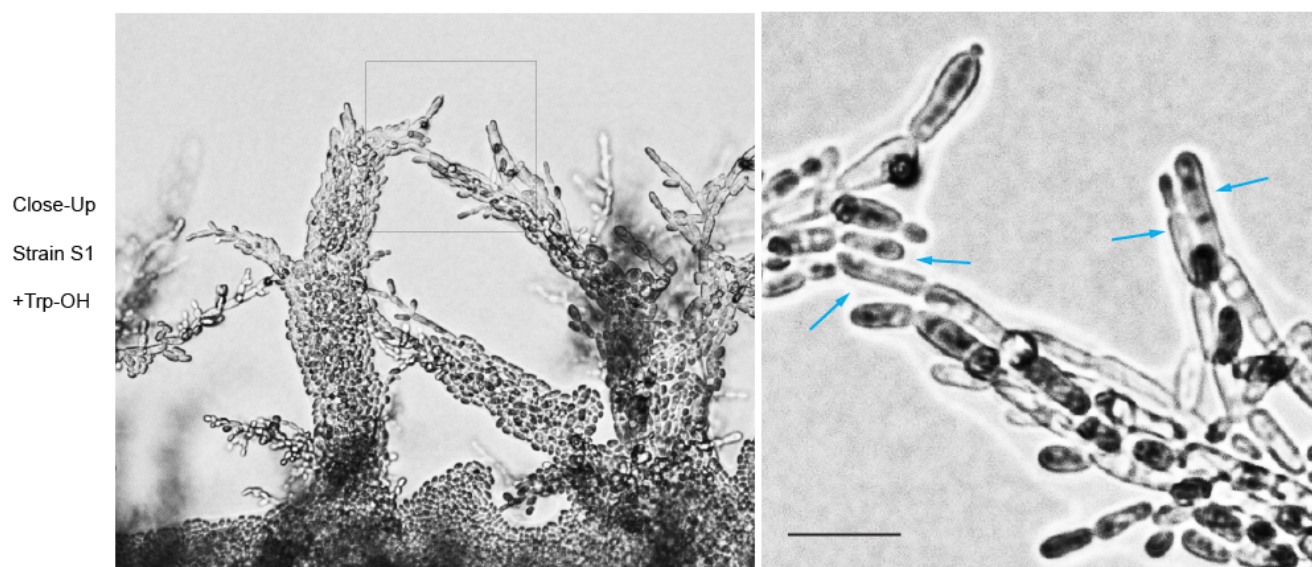

Supplement: FIG S6 [file mSphere.00702-18-sf006.pdf]

**Fig.\_S7**

**A**

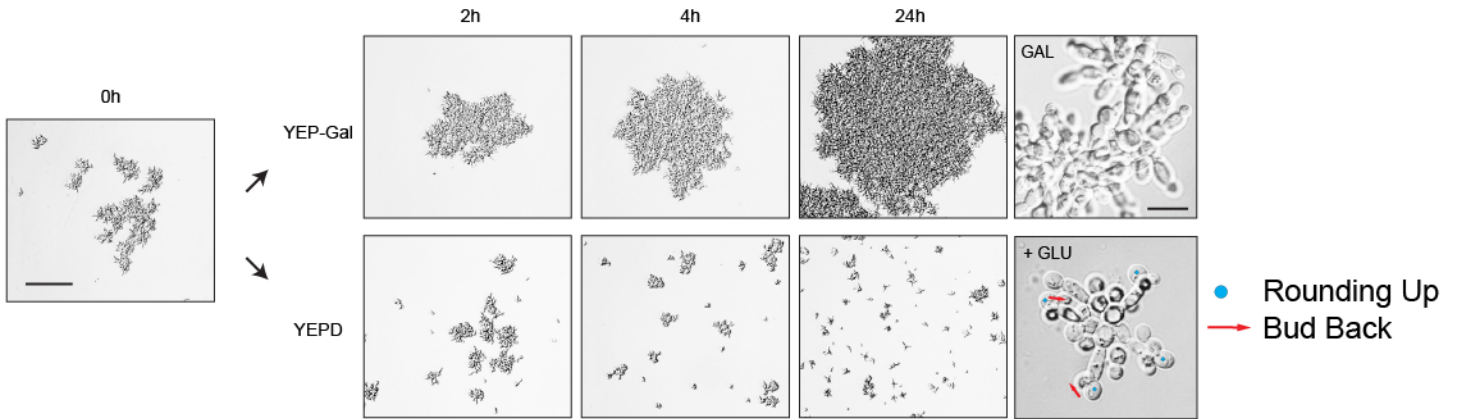

**B**

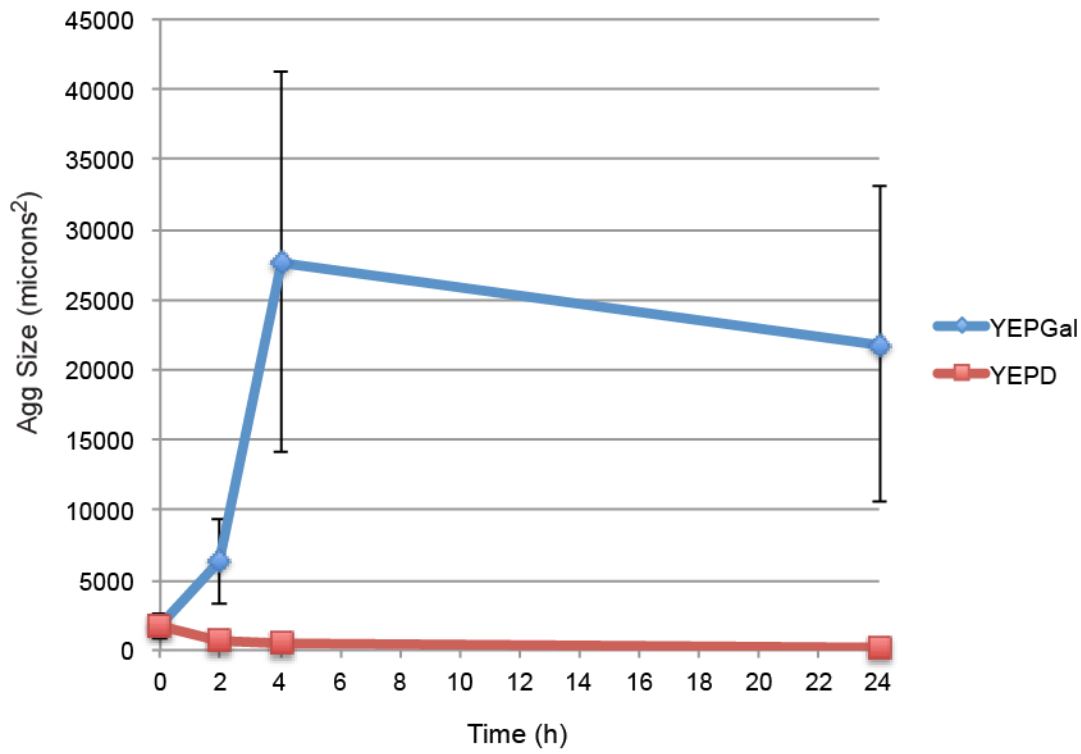

**C**

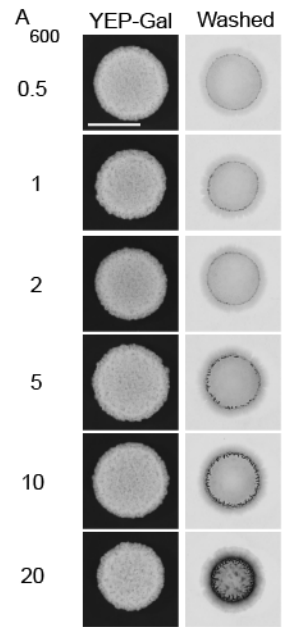

Supplement: FIG S7 [file mSphere.00702-18-sf007.pdf]

**A**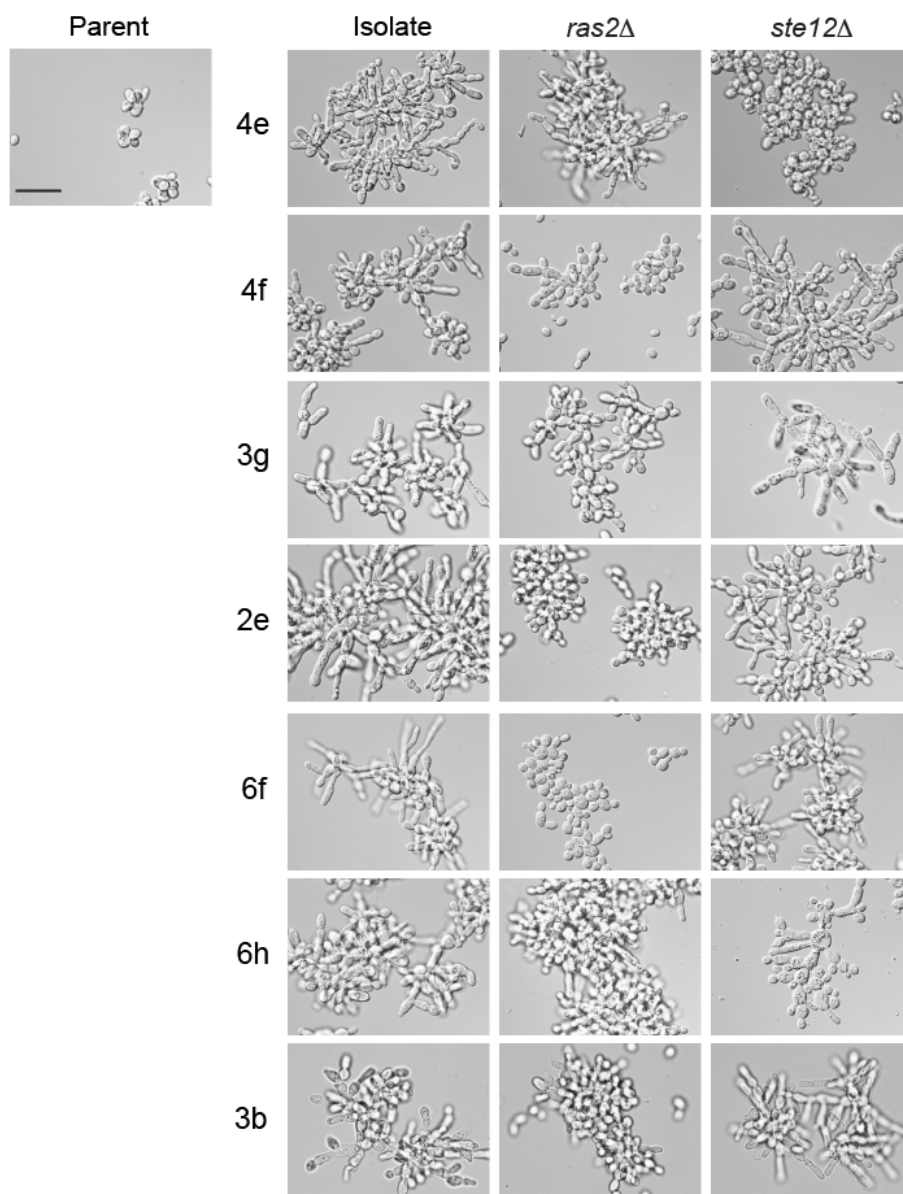**B**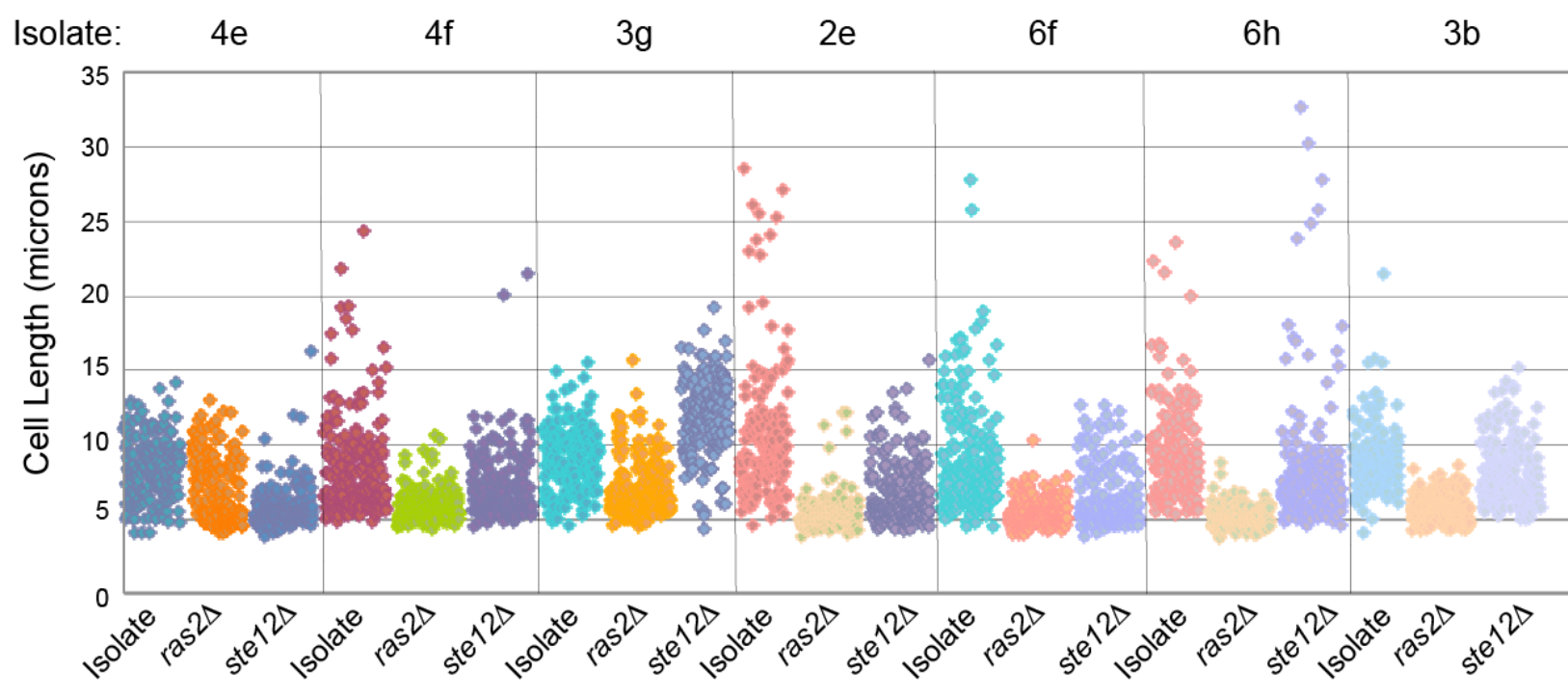

Supplement: FIG S8 [file mSphere.00702-18-sf008.pdf]
